# Supplementary material for: A systematic overexpression screen identifies cytotoxic genes encoded by the Cluster L1 mycobacteriophage LeBron
Source: G3 (Bethesda). 2026 Feb 19;16(5):jkag045. doi: 10.1093/g3journal/jkag045 (PMC13148394; doi:10.1093/g3journal/jkag045)
Supplement: jkag045_Supplementary_Data [file jkag045_supplementary_data.zip › Supplementary_Figure_1_G3-2025-406480.pdf]

Gene *1*; Score *0*

Images taken after 5 days at 37 ºC

Dilution factor: 10^{0}, 10^{-1}, 10^{-2}, 10^{-3}, 10^{-4}, 10^{-5}

Lanes: - + 1 2 3

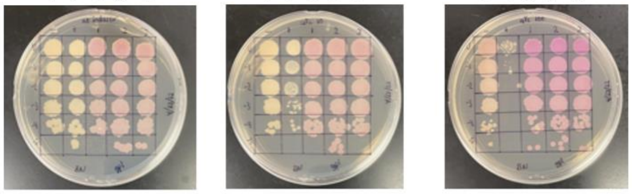

0 ng/ml aTc | 10 ng/ml aTc | 100 ng/ml aTc

Lane	Gene ID	Plasmid name	Gene name	Toxic/Non-toxic	Colony color on 100 ng/ml aTc plate*
+ Toxic control	--	pExTra02	Fruitloop *52*	Toxic	-
- Non-toxic control	--	pExTra03	Fruitloop *52* mutant	Non-toxic	+
1		pExTra-Lebron 1	Lebron *1* replicate 1	Non-toxic	+++
2		pExTra-Lebron 1	Lebron *1* replicate 2	Non-toxic	+++
3		pExTra-Lebron 1	Lebron *1* replicate 3	Non-toxic	+++

*Key: NG (no growth) - (no pink color) +(faint pink color) ++(obvious pink color) +++ (dark pink color)

Gene *2*; Score *0*

Images taken after 5 days at 37 ºC

Dilution factor: 10^{0}, 10^{-1}, 10^{-2}, 10^{-3}, 10^{-4}, 10^{-5}

Lanes: - + 1 2 3 | - + 1 2 3 | + - 1 2 3

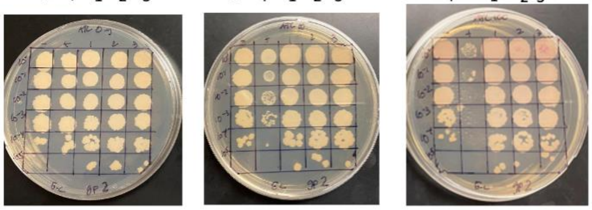

0 ng/ml aTc | 10 ng/ml aTc | 100 ng/ml aTc

Lane	Gene ID	Plasmid name	Gene name	Toxic/Non-toxic	Colony color on 100 ng/ml aTc plate*
+ Toxic control	--	pExTra02	Fruitloop *52*	Toxic	-
- Non-toxic control	--	pExTra03	Fruitloop *52* mutant	Non-toxic	+
1		pExTra-Lebron 2	Lebron 2 replicate 1	Non-toxic	++
2		pExTra-Lebron 2	Lebron 2 replicate 2	Non-toxic	++
3		pExTra-Lebron 2	Lebron 2 replicate 3	Non-toxic	++

*Key: NG (no growth) - (no pink color) +(faint pink color) ++(obvious pink color) +++ (dark pink color)

Gene *3*; Score *0*

Images taken after 5 days at 37 ºC

Dilution factor: 10^{0}, 10^{-1}, 10^{-2}, 10^{-3}, 10^{-4}, 10^{-5}

Lanes: - + 1 2 3

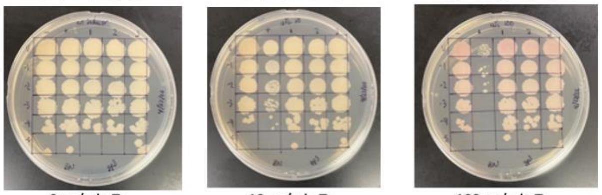

0 ng/ml aTc | 10 ng/ml aTc | 100 ng/ml aTc

Lane	Gene ID	Plasmid name	Gene name	Toxic/Non-toxic	Colony color on 100 ng/ml aTc plate*
+ Toxic control	--	pExTra02	Fruitloop *52*	Toxic	-
- Non-toxic control	--	pExTra03	Fruitloop *52* mutant	Non-toxic	+
1		pExTra-Lebron 3	Lebron *3* replicate 1	Non-toxic	+
2		pExTra-Lebron 3	Lebron *3* replicate 2	Non-toxic	+
3		pExTra-Lebron 3	Lebron *3* replicate 3	Non-toxic	+

*Key: NG (no growth) - (no pink color) +(faint pink color) ++(obvious pink color) +++ (dark pink color)

Gene *4*; Score *0*

Images taken after 5 days at 37 ºC

Dilution factor: 10^{0}, 10^{-1}, 10^{-2}, 10^{-3}, 10^{-4}, 10^{-5}

Lanes: - + 1 2 3

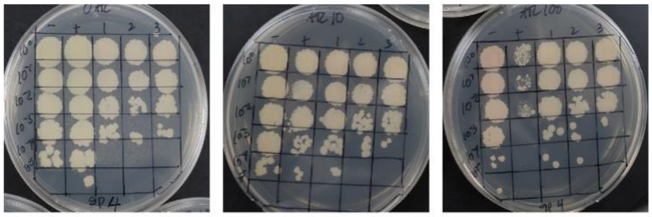

0 ng/ml aTc | 10 ng/ml aTc | 100 ng/ml aTc

Lane	Gene ID	Plasmid name	Gene name	Toxic/Non-toxic	Colony color on 100 ng/ml aTc plate*
+ Toxic control	--	pExTra02	Fruitloop *52*	Toxic	-
- Non-toxic control	--	pExTra03	Fruitloop *52* mutant	Non-toxic	+
1		pExTra-Lebron 4	Lebron *4* replicate 1	Non-toxic	+
2		pExTra-Lebron 4	Lebron *4* replicate 2	Non-toxic	+
3		pExTra-Lebron 4	Lebron *4* replicate 3	Non-toxic	+

*Key: NG (no growth) - (no pink color) +(faint pink color) ++(obvious pink color) +++ (dark pink color)

Gene *5*; Score *1*

Images taken after 5 days at 37 ºC

Dilution factor: 10^{0}, 10^{-1}, 10^{-2}, 10^{-3}, 10^{-4}, 10^{-5}

Lanes: - + 1 2 3

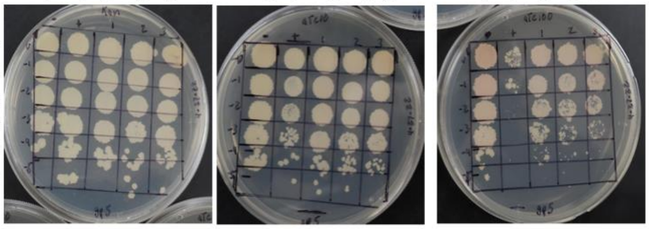

0 ng/ml aTc | 10 ng/ml aTc | 100 ng/ml aTc

Lane	Gene ID	Plasmid name	Gene name	Toxic/Non-toxic	Colony color on 100 ng/ml aTc plate*
+ Toxic control	--	pExTra02	Fruitloop *52*	Toxic	-
- Non-toxic control	--	pExTra03	Fruitloop *52* mutant	Non-toxic	+
1	131436	pExTra-Lebron 5	Lebron *5* replicate 1	Toxic	++
2	131436	pExTra-Lebron 5	Lebron *5* replicate 2	Toxic	++
3	131436	pExTra-Lebron 5	Lebron *5* replicate 3	Toxic	++

*Key: NG (no growth) - (no pink color) +(faint pink color) ++(obvious pink color) +++ (dark pink color)

Gene *6*; Score *0*

Images taken after 5 days at 37 ºC

Dilution factor: 10^{0}, 10^{-1}, 10^{-2}, 10^{-3}, 10^{-4}, 10^{-5}

Lanes: - + 1 2 3

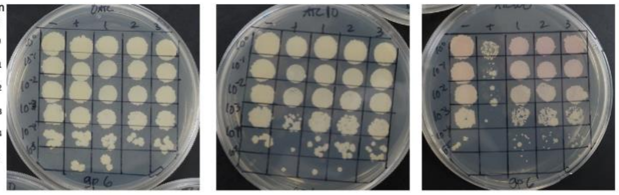

0 ng/ml aTc | 10 ng/ml aTc | 100 ng/ml aTc

Lane	Gene ID	Plasmid name	Gene name	Toxic/Non-toxic	Colony color on 100 ng/ml aTc plate*
- Non-toxic control	--	pExTra03	Fruitloop *52* mutant	Non-toxic	+
+ Toxic control	--	pExTra02	Fruitloop *52*	Toxic	-
1		pExTra-Lebron 6	Lebron *6* replicate 1	Non-toxic	++
2		pExTra-Lebron 6	Lebron *6* replicate 2	Non-toxic	++
3		pExTra-Lebron 6	Lebron *6* replicate 3	Non-toxic	++

*Key: NG (no growth) - (no pink color) +(faint pink color) ++(obvious pink color) +++ (dark pink color)

Gene *7*; Score *1*

Images taken after 5 days at 37 ºC

Dilution factor: 10^{0}, 10^{-1}, 10^{-2}, 10^{-3}, 10^{-4}, 10^{-5}

Lanes: - + 1 2 3

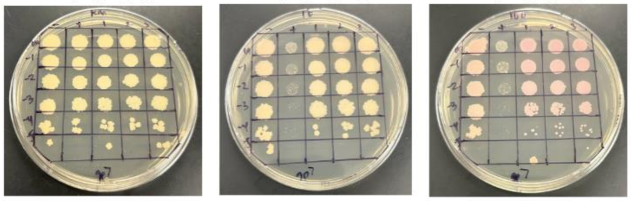

0 ng/ml aTc | 10 ng/ml aTc | 100 ng/ml aTc

Lane	Gene ID	Plasmid name	Gene name	Toxic/Non-toxic	Colony color on 100 ng/ml aTc plate*
- Non-toxic control	--	pExTra03	Fruitloop *52* mutant	Non-toxic	+
+ Toxic control	--	pExTra02	Fruitloop *52*	Toxic	-
1		pExTra-Lebron 7	Lebron *7* replicate 1	Toxic	++
2		pExTra-Lebron 7	Lebron *7* replicate 2	Toxic	++
3		pExTra-Lebron 7	Lebron *7* replicate 3	Toxic	++

*Key: NG (no growth) - (no pink color) +(faint pink color) ++(obvious pink color) +++ (dark pink color)

Gene *8*; Score *0*

Images taken after 5 days at 37 ºC

Dilution factor: 10^{0}, 10^{-1}, 10^{-2}, 10^{-3}, 10^{-4}, 10^{-5}

Lanes: - + 1 2 3

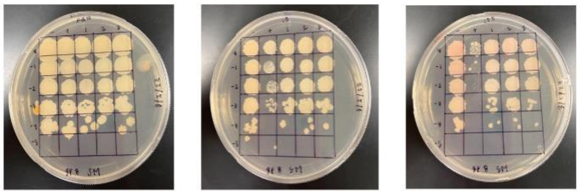

0 ng/ml aTc | 10 ng/ml aTc | 100 ng/ml aTc

Lane	Gene ID	Plasmid name	Gene name	Toxic/Non-toxic	Colony color on 100 ng/ml aTc plate*
+ Toxic control	--	pExTra02	Fruitloop *52*	Toxic	-
- Non-toxic control	--	pExTra03	Fruitloop *52* mutant	Non-toxic	+
1		pExTra-Lebron 8	Lebron *8* replicate 1	Non-Toxic	+
2		pExTra-Lebron 8	Lebron *8* replicate 2	Non-Toxic	+
3		pExTra-Lebron 8	Lebron 8 replicate 3	Non-Toxic	+

*Key: NG (no growth) - (no pink color) +(faint pink color) ++(obvious pink color) +++ (dark pink color)

Gene *9*; Score *0*

Images taken after 5 days at 37 ºC

\- + 1 2 3 | - + 1 2 3 | - + 1 2 3

Dilution factor: 10^0, 10^{-1}, 10^{-2}, 10^{-3}, 10^{-4}, 10^{-5}

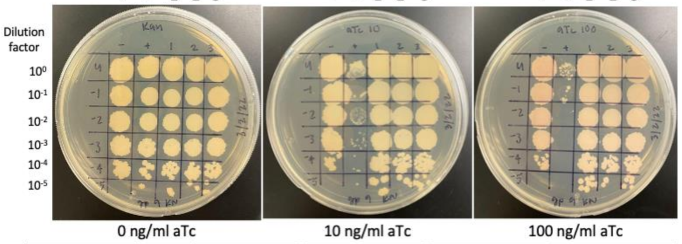

0 ng/ml aTc | 10 ng/ml aTc | 100 ng/ml aTc

Lane	Gene ID	Plasmid name	Gene name	Toxic/Non-toxic	Colony color on 100 ng/ml aTc plate*
- Non-toxic control	--	pExTra03	Fruitloop *52* mutant	Non-toxic	+
+ Toxic control	--	pExTra02	Fruitloop *52*	Toxic	-
1		pExTra-Lebron 9	Lebron 9 replicate 1	Non-toxic	+
2		pExTra-Lebron 9	Lebron 9 replicate 2	Non-toxic	+
3		pExTra-Lebron 9	Lebron 9 replicate 3	Non-toxic	+

*Key: NG (no growth) - (no pink color) +(faint pink color) ++(obvious pink color) +++ (dark pink color)

Gene *10*; Score *0*

Images taken after 5 days at 37 ºC

\- + 1 2 3 | - + 1 2 3 | - + 1 2 3

Dilution factor: 10^0, 10^{-1}, 10^{-2}, 10^{-3}, 10^{-4}, 10^{-5}

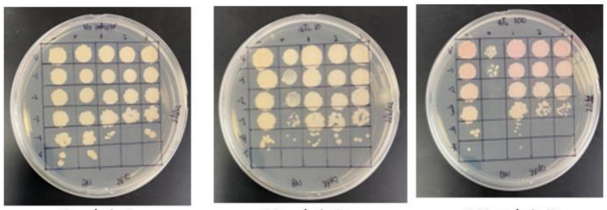

0 ng/ml aTc | 10 ng/ml aTc | 100 ng/ml aTc

Lane	Gene ID	Plasmid name	Gene name	Toxic/Non-toxic	Colony color on 100 ng/ml aTc plate*
- Non-toxic control	--	pExTra03	Fruitloop *52* mutant	Non-toxic	+
+ Toxic control	--	pExTra02	Fruitloop *52*	Toxic	+
1		pExTra-Lebron 10	Lebron *10* replicate 1	Non-toxic	++
2		pExTra-Lebron 10	Lebron *10* replicate 2	Non-toxic	++
3		pExTra-Lebron 10	Lebron *10* replicate 3	Non-toxic	++

*Key: NG (no growth) - (no pink color) +(faint pink color) ++(obvious pink color) +++ (dark pink color)

Gene *11*; Score *0*

Images taken after 5 days at 37 ºC

\+ - 1 2 3 | + - 1 2 3 | + - 1 2 3

Dilution factor: 10^0, 10^{-1}, 10^{-2}, 10^{-3}, 10^{-4}, 10^{-5}

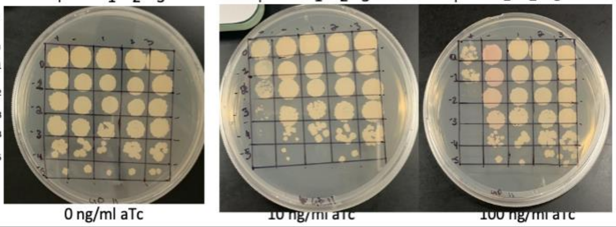

0 ng/ml aTc | 10 ng/ml aTc | 100 ng/ml aTc

Lane	Gene ID	Plasmid name	Gene name	Toxic/Non-toxic	Colony color on 100 ng/ml aTc plate*
+ Toxic control	--	pExTra02	Fruitloop *52*	Toxic	-
- Non-toxic control	--	pExTra03	Fruitloop *52* mutant	Non-toxic	++
1		pExTra-Lebron 11	Lebron *11* replicate 1	Non-toxic	-
2		pExTra-Lebron 11	Lebron *11* replicate 2	Non-toxic	-
3		pExTra-Lebron 11	Lebron *11* replicate 3	Non-toxic	-

*Key: NG (no growth) - (no pink color) +(faint pink color) ++(obvious pink color) +++ (dark pink color)

Gene *12*; Score *0*

Images taken after 5 days at 37 ºC

\- + 1 2 3 | - + 1 2 3 | - + 1 2 3

Dilution factor: 10^0, 10^{-1}, 10^{-2}, 10^{-3}, 10^{-4}, 10^{-5}

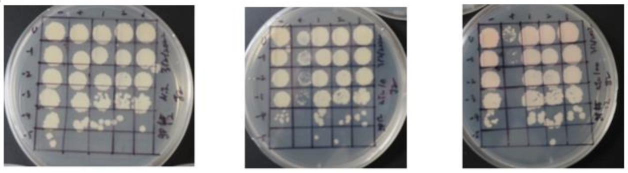

0 ng/ml aTc | 10 ng/ml aTc | 100 ng/ml aTc

Lane	Gene ID	Plasmid name	Gene name	Toxic/Non-toxic	Colony color on 100 ng/ml aTc plate*
- Non-toxic control	--	pExTra03	Fruitloop *52* mutant	Non-toxic	+
+ Toxic control	--	pExTra02	Fruitloop *52*	Toxic	-
1		pExTra-Lebron 12	Lebron *12* replicate 1	Non-toxic	+
2		pExTra-Lebron 12	Lebron *12* replicate 2	Non-toxic	+
3		pExTra-Lebron 12	Lebron *12* replicate 3	Non-toxic	+

*Key: NG (no growth) - (no pink color) +(faint pink color) ++(obvious pink color) +++ (dark pink color)

Gene *13*; Score *0*

Images taken after 5 days at 37 ºC

\- + 1 2 3 | - + 1 2 3 | - + 1 2 3

Dilution factor: 10^{-1}, 10^{-2}, 10^{-3}, 10^{-4}, 10^{-5}, 10^0

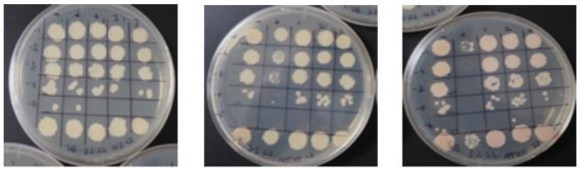

0 ng/ml aTc | 10 ng/ml aTc | 100 ng/ml aTc

Lane	Gene ID	Plasmid name	Gene name	Toxic/Non-toxic	Colony color on 100 ng/ml aTc plate*
+ Toxic control	--	pExTra02	Fruitloop *52*	Non-toxic	+
- Non-toxic control	--	pExTra03	Fruitloop *52* mutant	Toxic	-
1		pExTra-Lebron 13	Lebron 13 replicate 1	Non-toxic	+
2		pExTra-Lebron 13	Lebron 13 replicate 2	Non-toxic	+
3		pExTra-Lebron 13	Lebron 13 replicate 3	Non-toxic	+

*Key: NG (no growth) - (no pink color) +(faint pink color) ++(obvious pink color) +++ (dark pink color)

Gene *14*; Score *0*

Images taken after 5 days at 37 ºC

\- + 1 2 3 | - + 1 2 3 | + - 1 2 3

Dilution factor: 10^0, 10^{-1}, 10^{-2}, 10^{-3}, 10^{-4}, 10^{-5}

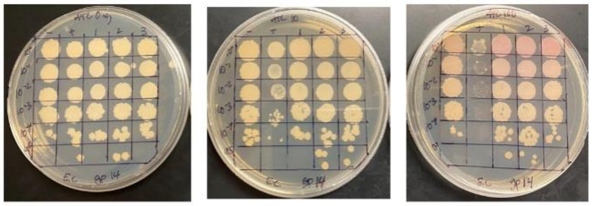

0 ng/ml aTc | 10 ng/ml aTc | 100 ng/ml aTc

Lane	Gene ID	Plasmid name	Gene name	Toxic/Non-toxic	Colony color on 100 ng/ml aTc plate*
+ Toxic control	--	pExTra02	Fruitloop *52*	Toxic	-
- Non-toxic control	--	pExTra03	Fruitloop *52* mutant	Non-toxic	+
1		pExTra-Lebron 14	Lebron 14 replicate 1	Non-toxic	++
2		pExTra-Lebron 14	Lebron 14 replicate 2	Non-toxic	++
3		pExTra-Lebron 14	Lebron 14 replicate 3	Non-toxic	++

*Key: NG (no growth) - (no pink color) +(faint pink color) ++(obvious pink color) +++ (dark pink color)

Gene *15*; Score *0*

Images taken after 5 days at 37 ºC

\- + 1 2 3 | - + 1 2 3 | - + 1 2 3

Dilution factor: 10^0, 10^{-1}, 10^{-2}, 10^{-3}, 10^{-4}, 10^{-5}

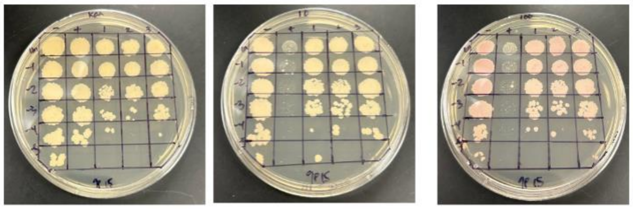

0 ng/ml aTc | 10 ng/ml aTc | 100 ng/ml aTc

Lane	Gene ID	Plasmid name	Gene name	Toxic/Non-toxic	Colony color on 100 ng/ml aTc plate*
- Non-toxic control	--	pExTra03	Fruitloop *52* mutant	Non-toxic	++
+ Toxic control	--	pExTra02	Fruitloop *52*	Toxic	-
1		pExTra-Lebron 15	Lebron *15* replicate 1	Non-toxic	+
2		pExTra-Lebron 15	Lebron *15* replicate 2	Non-toxic	+
3		pExTra-Lebron 15	Lebron *15* replicate 3	Non-toxic	+

*Key: NG (no growth) - (no pink color) +(faint pink color) ++(obvious pink color) +++ (dark pink color)

Gene *16*; Score *0*

Images taken after 5 days at 37 ºC

\- + 1 2 3 | - + 1 2 3 | - + 1 2 3

Dilution factor: 10^0, 10^{-1}, 10^{-2}, 10^{-3}, 10^{-4}, 10^{-5}

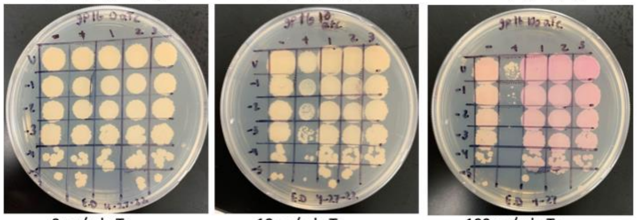

0 ng/ml aTc | 10 ng/ml aTc | 100 ng/ml aTc

Lane	Gene ID	Plasmid name	Gene name	Toxic/Non-toxic	Colony color on 100 ng/ml aTc plate*
+ Toxic control	--	pExTra02	Fruitloop *52*	Toxic	-
- Non-toxic control	--	pExTra03	Fruitloop *52* mutant	Non-toxic	++
1		pExTra-Lebron 16	Lebron *16* replicate 1	Non-toxic	+++
2		pExTra-Lebron 16	Lebron *16* replicate 2	Non-toxic	+++
3		pExTra-Lebron 16	Lebron *16* replicate 3	Non-toxic	+++

*Key: NG (no growth) - (no pink color) +(faint pink color) ++(obvious pink color) +++ (dark pink color)

Gene *17*; Score *0*

Images taken after 5 days at 37 ºC

- + 1 2 3   - + 1 2 3   - + 1 2 3

Dilution factor: 10^0, 10^{-1}, 10^{-2}, 10^{-3}, 10^{-4}, 10^{-5}

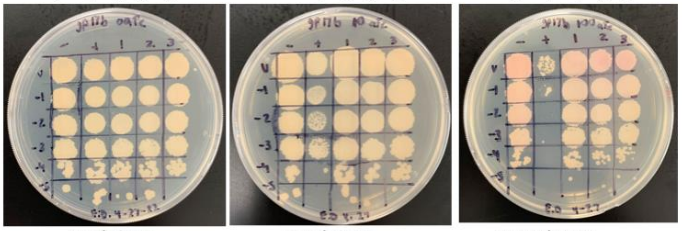

0 ng/ml aTc   10 ng/ml aTc   100 ng/ml aTc

Lane	Gene ID	Plasmid name	Gene name	Toxic/Non-toxic	Colony color on 100 ng/ml aTc plate*
+ Toxic control	--	pExTra02	Fruitloop *52*	Toxic	-
- Non-toxic control	--	pExTra03	Fruitloop *52* mutant	Non-toxic	+
1		pExTra-LeBron 17	LeBron *17* replicate 1	Non-toxic	+
2		pExTra-LeBron 17	LeBron *17* replicate 2	Non-toxic	+
3		pExTra-LeBron 17	LeBron *17* replicate 3	Non-toxic	+

*Key: NG (no growth)   - (no pink color)   +(faint pink color)   ++(obvious pink color)   +++ (dark pink color)

Gene *18*; Score *0*

Images taken after 5 days at 37 ºC

- + 1 2 3   - + 1 2 3   - + 1 2 3

Dilution factor: 10^0, 10^{-1}, 10^{-2}, 10^{-3}, 10^{-4}, 10^{-5}

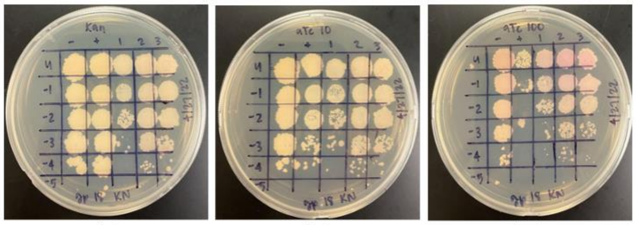

0 ng/ml aTc   10 ng/ml aTc   100 ng/ml aTc

Lane	Gene ID	Plasmid name	Gene name	Toxic/Non-toxic	Colony color on 100 ng/ml aTc plate*
- Non-toxic control	--	pExTra03	Fruitloop 52 mutant	Non-toxic	+
+ Toxic control	--	pExTra02	Fruitloop *52*	Toxic	-
1		pExTra-Lebron 18	Lebron 18 replicate 1	Non-toxic	++
2		pExTra-Lebron 18	Lebron 18 replicate 2	Non-toxic	++
3		pExTra-Lebron 18	Lebron 18 replicate 3	Non-toxic	++

*Key: NG (no growth)   - (no pink color)   +(faint pink color)   ++(obvious pink color)   +++ (dark pink color)

Gene *19*; Score *0*

Images taken after 5 days at 37 ºC

- + 1 2 3   - + 1 2 3   - + 1 2 3

Dilution factor: 10^0, 10^{-1}, 10^{-2}, 10^{-3}, 10^{-4}, 10^{-5}

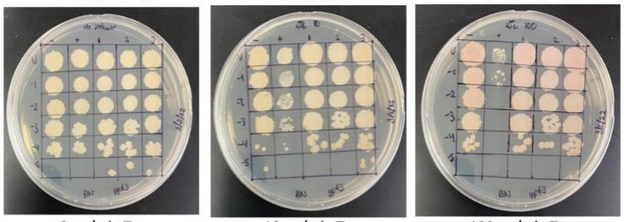

0 ng/ml aTc   10 ng/ml aTc   100 ng/ml aTc

Lane	Gene ID	Plasmid name	Gene name	Toxic/Non-toxic	Colony color on 100 ng/ml aTc plate*
- Non-toxic control	--	pExTra03	Fruitloop 52 mutant	Non-toxic	++
+ Toxic control	--	pExTra02	Fruitloop *52*	Toxic	+
1		pExTra-Lebron 19	Lebron *19* replicate 1	Non-toxic	++
2		pExTra-Lebron 19	Lebron *19* replicate 2	Non-toxic	++
3		pExTra-Lebron 19	Lebron *19* replicate 3	Non-toxic	++

*Key: NG (no growth)   - (no pink color)   +(faint pink color)   ++(obvious pink color)   +++ (dark pink color)

Gene *20*; Score *1*

Images taken after 5 days at 37 ºC

- + 1 2 3   - + 1 2 3   - + 1 2 3

Dilution factor: 10^0, 10^{-1}, 10^{-2}, 10^{-3}, 10^{-4}, 10^{-5}

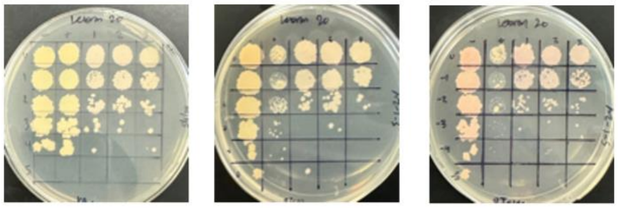

0 ng/ml aTc   10 ng/ml aTc   100 ng/ml aTc

Lane	Gene ID	Plasmid name	Gene name	Toxic/Non-toxic	Colony color on 100 ng/ml aTc plate*
- Non-toxic control	--	pExTra03	Fruitloop *52* mutant	Non-toxic	+
+ Toxic control	--	pExTra02	Fruitloop *52*	Toxic	-
1		pExTra-Lebron 20	Lebron *20* replicate 1	Toxic	++
2		pExTra-Lebron 20	Lebron *20* replicate 2	Toxic	++
3		pExTra-Lebron 20	Lebron *20* replicate 3	Toxic	++

*Key: NG (no growth)   - (no pink color)   +(faint pink color)   ++(obvious pink color)   +++ (dark pink color)

Gene *21*; Score *0*

Images taken after 5 days at 37 ºC

- + 1 2 3   - + 1 2 3   - + 1 2 3

Dilution factor: 10^0, 10^{-1}, 10^{-2}, 10^{-3}, 10^{-4}, 10^{-5}

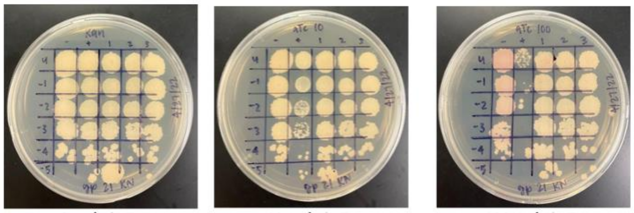

0 ng/ml aTc   10 ng/ml aTc   100 ng/ml aTc

Lane	Gene ID	Plasmid name	Gene name	Toxic/Non-toxic	Colony color on 100 ng/ml aTc plate*
- Non-toxic control	--	pExTra03	Fruitloop *52* mutant	Non-toxic	+
+ Toxic control	--	pExTra02	Fruitloop *52*	Toxic	-
1		pExTra-Lebron 21	Lebron 21 replicate 1	Non-toxic	-
2		pExTra-Lebron 21	Lebron 21 replicate 2	Non-toxic	-
3		pExTra-Lebron 21	Lebron 21 replicate 3	Non-toxic	-

*Key: NG (no growth)   - (no pink color)   +(faint pink color)   ++(obvious pink color)   +++ (dark pink color)

Gene *22*; Score *0*

Images taken after 5 days at 37 ºC

- + 1 2 3   - + 1 2 3   - + 1 2 3

Dilution factor: 10^0, 10^{-1}, 10^{-2}, 10^{-3}, 10^{-4}, 10^{-5}

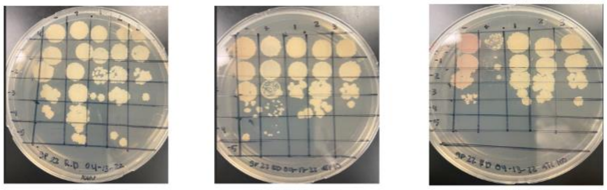

0 ng/ml aTc   10 ng/ml aTc   100 ng/ml aTc

Lane II	Gene ID	Plasmid name	Gene name	Toxic/Non-toxic	Colony color on 100 ng/ml aTc plate*
- Non-toxic control	--	pExTra03	Fruitloop *52 mutant*	Non-toxic	++
+ Toxic control	--	pExTra02	Fruitloop *52*	Toxic	+
1		pExTra-Lebron 22	Lebron 22 replicate 1	Non-toxic	-
2		pExTra-Lebron 22	Lebron 22 replicate 2	Non-toxic	-
3		pExTra-Lebron 22	Lebron 22 replicate 3	Non-toxic	-

*Key: NG (no growth)   - (no pink color)   +(faint pink color)   ++(obvious pink color)   +++ (dark pink color)

Gene *23*; Score *3*

Images taken after 5 days at 37 ºC

- + 1 2 3   - + 1 2 3   - + 1 2 3

Dilution factor: 10^0, 10^{-1}, 10^{-2}, 10^{-3}, 10^{-4}, 10^{-5}

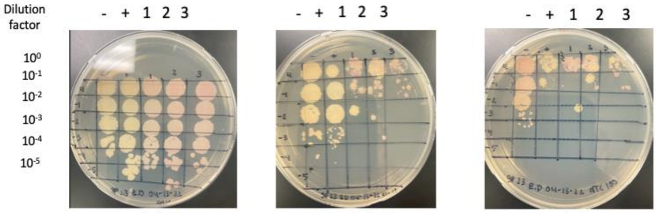

0 ng/ml aTc   10 ng/ml aTc   100 ng/ml aTc

Lane II	Gene ID	Plasmid name	Gene name	Toxic/Non-toxic	Colony color on 100 ng/ml aTc plate*
- Non-toxic control	--	pExTra03	Fruitloop *52 mutant*	Non-toxic	+++
+ Toxic control	--	pExTra02	Fruitloop *52*	Toxic	+
1		pExTra-Lebron 23	Lebron 23 replicate 1	Toxic	+++
2		pExTra-Lebron 23	Lebron 23 replicate 2	Toxic	+
3		pExTra-Lebron 23	Lebron 23 replicate 3	Toxic	++

*Key: NG (no growth)   - (no pink color)   +(faint pink color)   ++(obvious pink color)   +++ (dark pink color)

Gene *24*; Score *0*

Images taken after 5 days at 37 ºC

- + 1 2 3   - + 1 2 3   - + 1 2 3

Dilution factor: 10^0, 10^{-1}, 10^{-2}, 10^{-3}, 10^{-4}, 10^{-5}

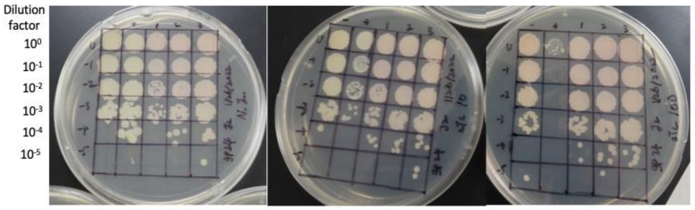

0 ng/ml aTc   10 ng/ml aTc   100 ng/ml aTc

Lane	Gene ID	Plasmid name	Gene name	Toxic/Non-toxic	Colony color on 100 ng/ml aTc plate*
- Non-toxic control	--	pExTra03	Fruitloop 52 mutant	Non-toxic	+
+ Toxic control	--	pExTra02	Fruitloop 52	Toxic	-
1		pExTra-Lebron 24	Lebron *24* replicate 1	Non-toxic	+
2		pExTra-Lebron 24	Lebron *24* replicate 2	Non-toxic	+
3		pExTra-Lebron 24	Lebron *24* replicate 3	Non-toxic	+

*Key: NG (no growth)   - (no pink color)   +(faint pink color)   ++(obvious pink color)   +++ (dark pink color)

Gene *25*; Score *2*

Images taken after 5 days at 37 ºC

Dilution factor: 10^0, 10^-1, 10^-2, 10^-3, 10^-4, 10^-5 — Lanes: - + 1 2 3

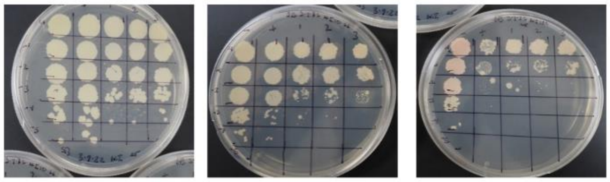

0 ng/ml aTc | 10 ng/ml aTc | 100 ng/ml aTc

Lane	Gene ID	Plasmid name	Gene name	Toxic/Non-toxic	Colony color on 100 ng/ml aTc plate*
+ Toxic control	--	pExTra02	Fruitloop *52*	Non-toxic	+
- Non-toxic control	--	pExTra03	Fruitloop *52* mutant	Toxic	-
1	131436	pExTra-Lebron 25	Lebron 25 replicate 1	Toxic	-
2	131436	pExTra-Lebron 25	Lebron 25 replicate 2	Toxic	-
3	131436	pExTra-Lebron 25	Lebron 25 replicate 3	Toxic	-

*Key: NG (no growth) - (no pink color) +(faint pink color) ++(obvious pink color) +++ (dark pink color)

Gene *26*; Score *0*

Images taken after 5 days at 37 ºC

Dilution factor: 10^0, 10^-1, 10^-2, 10^-3, 10^-4, 10^-5 — Lanes: - + 1 2 3

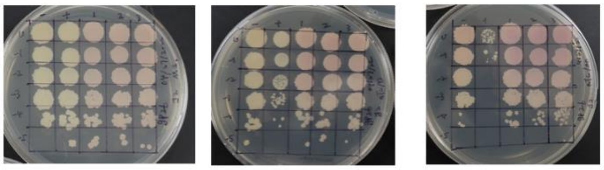

0 ng/ml aTc | 10 ng/ml aTc | 100 ng/ml aTc

Lane	Gene ID	Plasmid name	Gene name	Toxic/Non-toxic	Colony color on 100 ng/ml aTc plate*
- Non-toxic control	--	pExTra03	Fruitloop *52* mutant	Non-toxic	+
+ Toxic control	--	pExTra02	Fruitloop *52*	Toxic	-
1		pExTra-Lebron 26	Lebron *26* replicate 1	Non-toxic	++
2		pExTra-Lebron 26	Lebron *26* replicate 2	Non-toxic	++
3		pExTra-Lebron 26	Lebron *26* replicate 3	Non-toxic	++

*Key: NG (no growth) - (no pink color) +(faint pink color) ++(obvious pink color) +++ (dark pink color)

Gene *27*; Score *0*

Images taken after 5 days at 37 ºC

Dilution factor: 10^0, 10^-1, 10^-2, 10^-3, 10^-4, 10^-5 — Lanes: - + 1 2 3

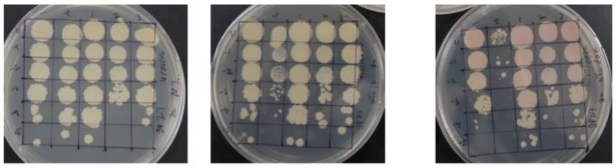

0 ng/ml aTc | 10 ng/ml aTc | 100 ng/ml aTc

Lane	Gene ID	Plasmid name	Gene name	Toxic/Non-toxic	Colony color on 100 ng/ml aTc plate*
- Non-toxic control	--	pExTra03	Fruitloop *52* mutant	Non-toxic	+
+ Toxic control	--	pExTra02	Fruitloop *52*	Toxic	-
1		pExTra-Lebron 27	Lebron *27* replicate 1	Non-toxic	+
2		pExTra-Lebron 27	Lebron *27* replicate 2	Non-toxic	+
3		pExTra-Lebron 27	Lebron *27* replicate 3	Non-toxic	+

*Key: NG (no growth) - (no pink color) +(faint pink color) ++(obvious pink color) +++ (dark pink color)

Gene *28*; Score *0*

Images taken after 5 days at 37 ºC

Dilution factor: 10^0, 10^-1, 10^-2, 10^-3, 10^-4, 10^-5 — Lanes: - + 1 2 3

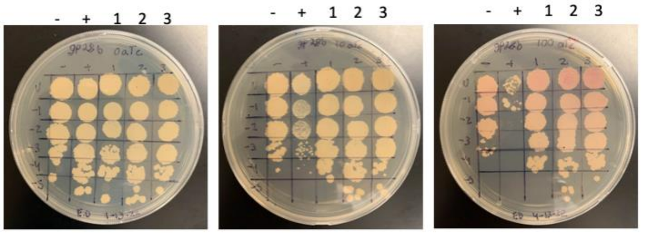

0 ng/ml aTc | 10 ng/ml aTc | 100 ng/ml aTc

Lane	Gene ID	Plasmid name	Gene name	Toxic/Non-toxic	Colony color on 100 ng/ml aTc plate*
+ Toxic control	--	pExTra02	Fruitloop *52*	Toxic	-
- Non-toxic control	--	pExTra03	Fruitloop *52* mutant	Non-toxic	+
1		pExTra-Lebron 28	Lebron *28* replicate 1	Non-toxic	++
2		pExTra-Lebron 28	Lebron *28* replicate 2	Non-toxic	++
3		pExTra-Lebron 28	Lebron *28* replicate 3	Non-toxic	++

*Key: NG (no growth) - (no pink color) +(faint pink color) ++(obvious pink color) +++ (dark pink color)

Gene *29*; Score *2*

Images taken after 5 days at 37 ºC

Dilution factor: 10^0, 10^-1, 10^-2, 10^-3, 10^-4, 10^-5 — Lanes: - + 1 2 3

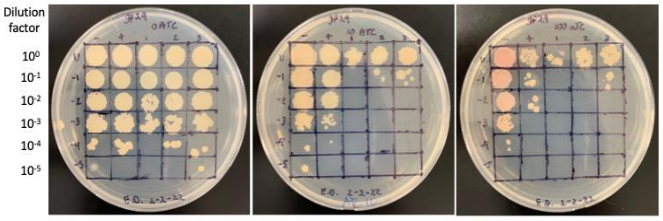

0 ng/ml aTc | 10 ng/ml aTc | 100 ng/ml aTc

Lane	Gene ID	Plasmid name	Gene name	Toxic/Non-toxic	Colony color on 100 ng/ml aTc plate*
+ Toxic control	--	pExTra02	Fruitloop *52*	Toxic	-
- Non-toxic control	--	pExTra03	Fruitloop *52* mutant	Non-toxic	++
1		pExTra-LeBron 29	LeBeon *29* replicate 1	Toxic	+
2		pExTra-LeBron 29	LeBeon *29* replicate 2	Toxic	-
3		pExTra-LeBron 29	LeBeon *29* replicate 3	Toxic	+

*Key: NG (no growth) - (no pink color) +(faint pink color) ++(obvious pink color) +++ (dark pink color)

Gene *30*; Score *0*

Images taken after 5 days at 37 ºC

Dilution factor: 10^0, 10^-1, 10^-2, 10^-3, 10^-4, 10^-5 — Lanes: - + 1 2 3

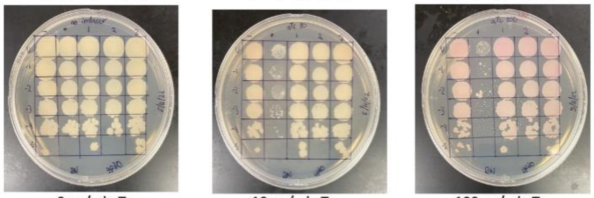

0 ng/ml aTc | 10 ng/ml aTc | 100 ng/ml aTc

Lane	Gene ID	Plasmid name	Gene name	Toxic/Non-toxic	Colony color on 100 ng/ml aTc plate*
+ Toxic control	--	pExTra02	Fruitloop *52*	Toxic	+
- Non-toxic control	--	pExTra03	Fruitloop *52* mutant	Non-toxic	+
1		pExTra-Lebron 30	Lebron *30* replicate 1	Non-toxic	++
2		pExTra-Lebron 30	Lebron *30* replicate 2	Non-toxic	++
3		pExTra-Lebron 30	Lebron *30* replicate 3	Non-toxic	++

*Key: NG (no growth) - (no pink color) +(faint pink color) ++(obvious pink color) +++ (dark pink color)

Gene *31*; Score *0*

Images taken after 5 days at 37 ºC

Dilution factor: 10^0, 10^-1, 10^-2, 10^-3, 10^-4, 10^-5 — Lanes: - + 1 2 3

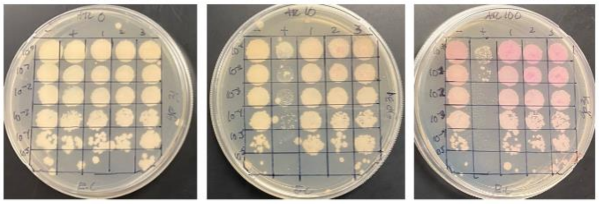

0 ng/ml aTc | 10 ng/ml aTc | 100 ng/ml aTc

Lane	Gene ID	Plasmid name	Gene name	Toxic/Non-toxic	Colony color on 100 ng/ml aTc plate*
+ Toxic control	--	pExTra02	Fruitloop *52*	Toxic	-
- Non-toxic control	--	pExTra03	Fruitloop *52* mutant	Non-toxic	+
1		pExTra-Lebron 31	Lebron 31 replicate 1	Non-toxic	++
2		pExTra-Lebron 31	Lebron 31 replicate 2	Non-toxic	++
3		pExTra-Lebron 31	Lebron 31 replicate 3	Non-toxic	++

*Key: NG (no growth) - (no pink color) +(faint pink color) ++(obvious pink color) +++ (dark pink color)

Gene *32*; Score *0*

Images taken after 5 days at 37 ºC

Dilution factor: 10^0, 10^-1, 10^-2, 10^-3, 10^-4, 10^-5 — Lanes: - + 1 2 3

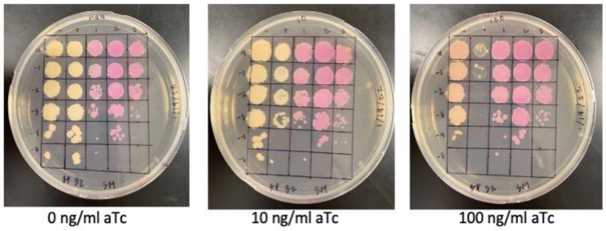

0 ng/ml aTc | 10 ng/ml aTc | 100 ng/ml aTc

Lane	Gene ID	Plasmid name	Gene name	Toxic/Non-toxic	Colony color on 100 ng/ml aTc plate*
+ Toxic control	--	pExTra02	Fruitloop *52*	Toxic	-
- Non-toxic control	--	pExTra03	Fruitloop *52* mutant	Non-toxic	+
1		pExTra-Lebron 32	Lebron *32* replicate 1	Non-toxic	+++
2		pExTra-Lebron 32	Lebron *32* replicate 2	Non-toxic	+++
3		pExTra-Lebron 32	Lebron *32* replicate 3	Non-toxic	+++

*Key: NG (no growth) - (no pink color) +(faint pink color) ++(obvious pink color) +++ (dark pink color)

Gene *33*; Score *0*

Images taken after 5 days at 37 ºC

- + 1 2 3 | - + 1 2 3 | - + 1 2 3

Dilution factor: 10^{0}, 10^{-1}, 10^{-2}, 10^{-3}, 10^{-4}, 10^{-5}

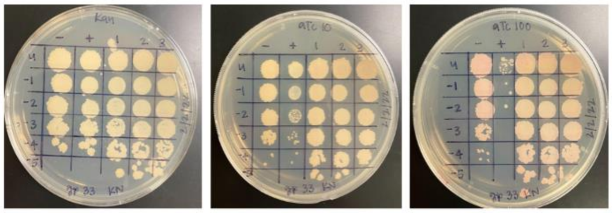

0 ng/ml aTc | 10 ng/ml aTc | 100 ng/ml aTc

Lane	Gene ID	Plasmid name	Gene name	Toxic/Non-toxic	Colony color on 100 ng/ml aTc plate*
- Non-toxic control	--	pExTra03	Fruitloop *52* mutant	Non-toxic	+
+ Toxic control	--	pExTra02	Fruitloop *52*	Toxic	-
1		pExTra-Lebron 33	Lebron 33 replicate 1	Non-toxic	+
2		pExTra-Lebron 33	Lebron 33 replicate 2	Non-toxic	+
3		pExTra-Lebron 33	Lebron 33 replicate 3	Non-toxic	+

*Key: NG (no growth) - (no pink color) +(faint pink color) ++(obvious pink color) +++ (dark pink color)

Gene *34*; Score *0*

Images taken after 5 days at 37 ºC

- + 1 2 3 | - + 1 2 3 | - + 1 2 3

Dilution factor: 10^{0}, 10^{-1}, 10^{-2}, 10^{-3}, 10^{-4}, 10^{-5}

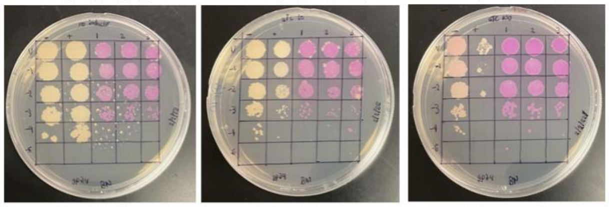

0 ng/ml aTc | 10 ng/ml aTc | 100 ng/ml aTc

Lane	Gene ID	Plasmid name	Gene name	Toxic/Non-toxic	Colony color on 100 ng/ml aTc plate*
- Non-toxic control	--	pExTra03	Fruitloop *52* mutant	Non-toxic	+
+ Toxic control	--	pExTra02	Fruitloop *52*	Toxic	-
1		pExTra-Lebron 34	Lebron *34* replicate 1	Non-toxic	+++
2		pExTra-Lebron 34	Lebron *34* replicate 2	Non-toxic	+++
3		pExTra-Lebron 34	Lebron *34* replicate 3	Non-toxic	+++

*Key: NG (no growth) - (no pink color) +(faint pink color) ++(obvious pink color) +++ (dark pink color)

Gene *35*; Score *3*

Images taken after 5 days at 37 ºC

- + 1 2 3 | - + 1 2 3 | - + 1 2 3

Dilution factor: 10^{0}, 10^{-1}, 10^{-2}, 10^{-3}, 10^{-4}, 10^{-5}

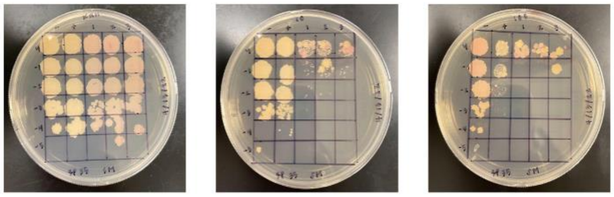

0 ng/ml aTc | 10 ng/ml aTc | 100 ng/ml aTc

Lane	Gene ID	Plasmid name	Gene name	Toxic/Non-toxic	Colony color on 100 ng/ml aTc plate*
+ Toxic control	--	pExTra02	Fruitloop *52*	Toxic	-
- Non-toxic control	--	pExTra03	Fruitloop *52* mutant	Non-toxic	+
1		pExTra-Lebron35	Lebron *35* replicate 1	Toxic	+
2		pExTra-Lebron35	Lebron *35* replicate 2	Toxic	+
3		pExTra-Lebron35	Lebron *35* replicate 3	Toxic	+

*Key: NG (no growth) - (no pink color) +(faint pink color) ++(obvious pink color) +++ (dark pink color)

Gene *36*; Score *1*

Images taken after 5 days at 37 ºC

- + 1 2 3 | - + 1 2 3 | - + 1 2 3

Dilution factor: 10^{0}, 10^{-1}, 10^{-2}, 10^{-3}, 10^{-4}, 10^{-5}

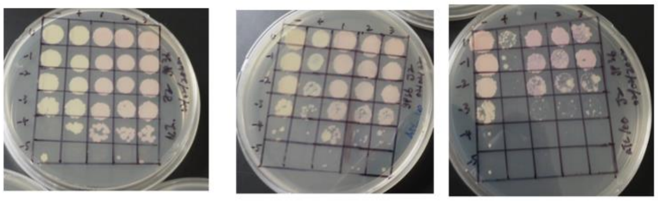

0 ng/ml aTc | 10 ng/ml aTc | 100 ng/ml aTc

Lane	Gene ID	Plasmid name	Gene name	Toxic/Non-toxic	Colony color on 100 ng/ml aTc plate*
- Non-toxic control	--	pExTra03	Fruitloop *52* mutant	Non-toxic	+
+ Toxic control	--	pExTra02	Fruitloop *52*	Toxic	-
1		pExTra-Lebron 36	Lebron *36* replicate 1	Toxic	++
2		pExTra-Lebron 36	Lebron *36* replicate 2	Toxic	++
3		pExTra-Lebron 36	Lebron *36* replicate 3	Toxic	++

*Key: NG (no growth) - (no pink color) +(faint pink color) ++(obvious pink color) +++ (dark pink color)

Gene *37*; Score *0*

Images taken after 5 days at 37 ºC

- + 1 2 3 | - + 1 2 3 | - + 1 2 3

Dilution factor: 10^{0}, 10^{-1}, 10^{-2}, 10^{-3}, 10^{-4}, 10^{-5}

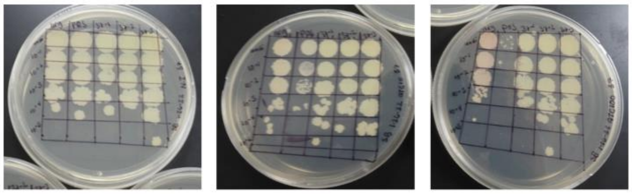

0 ng/ml aTc | 10 ng/ml aTc | 100 ng/ml aTc

Lane	Gene ID	Plasmid name	Gene name	Toxic/Non-toxic	Colony color on 100 ng/ml aTc plate*
+ Toxic control	--	pExTra02	Fruitloop *52*	Non-toxic	+
- Non-toxic control	--	pExTra03	Fruitloop *52* mutant	Toxic	-
1		pExTra-Lebron 37	Lebron 37 replicate 1	Non-toxic	-
2		pExTra-Lebron 37	Lebron 37 replicate 2	Non-toxic	-
3		pExTra-Lebron 37	Lebron 37 replicate 3	Non-toxic	-

*Key: NG (no growth) - (no pink color) +(faint pink color) ++(obvious pink color) +++ (dark pink color)

Gene *38*; Score *0*

Images taken after 5 days at 37 ºC

- + 1 2 3 | - + 1 2 3 | - + 1 2 3

Dilution factor: 10^{0}, 10^{-1}, 10^{-2}, 10^{-3}, 10^{-4}, 10^{-5}

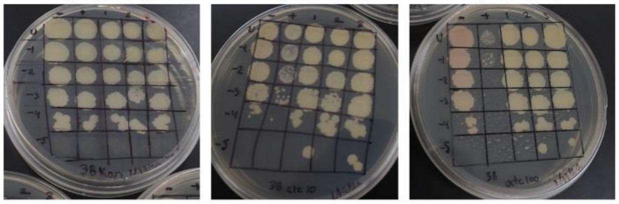

0 ng/ml aTc | 10 ng/ml aTc | 100 ng/ml aTc

Lane	Gene ID	Plasmid name	Gene name	Toxic/Non-toxic	Colony color on 100 ng/ml aTc plate*
- Non-toxic control	--	pExTra03	Fruitloop *52* mutant	Non-toxic	+
+ Toxic control	--	pExTra02	Fruitloop *52*	Toxic	-
1		pExTra-Lebron38	Lebron *38* replicate 1	Non-toxic	-
2		pExTra-Lebron 38	Lebron *38* replicate 2	Non-toxic	-
3		pExTra-Lebron 38	Lebron *38* replicate 3	Non-toxic	-

*Key: NG (no growth) - (no pink color) +(faint pink color) ++(obvious pink color) +++ (dark pink color)

Gene *39*; Score *0*

Images taken after 5 days at 37 ºC

- + 1 2 3 | - + 1 2 3 | - + 1 2 3

Dilution factor: 10^{0}, 10^{-1}, 10^{-2}, 10^{-3}, 10^{-4}, 10^{-5}

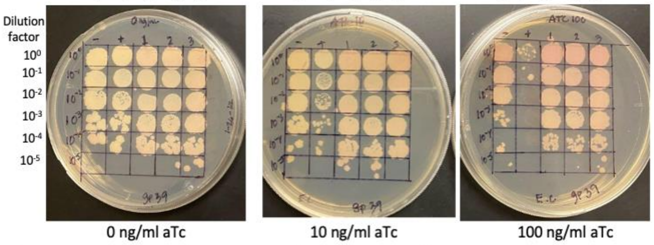

0 ng/ml aTc | 10 ng/ml aTc | 100 ng/ml aTc

Lane	Gene ID	Plasmid name	Gene name	Toxic/Non-toxic	Colony color on 100 ng/ml aTc plate*
+ Toxic control	--	pExTra02	Fruitloop *52*	Toxic	-
- Non-toxic control	--	pExTra03	Fruitloop *52* mutant	Non-toxic	+
1		pExTra-Lebron 39	Lebron 39 replicate 1	Non-toxic	++
2		pExTra-Lebron 39	Lebron 39 replicate 2	Non-toxic	++
3		pExTra-Lebron 39	Lebron *39* replicate 3	Non-toxic	++

*Key: NG (no growth) - (no pink color) +(faint pink color) ++(obvious pink color) +++ (dark pink color)

Gene *40*; Score *0*

Images taken after 5 days at 37 ºC

- + 1 2 3 | - + 1 2 3 | - + 1 2 3

Dilution factor: 10^{0}, 10^{-1}, 10^{-2}, 10^{-3}, 10^{-4}, 10^{-5}

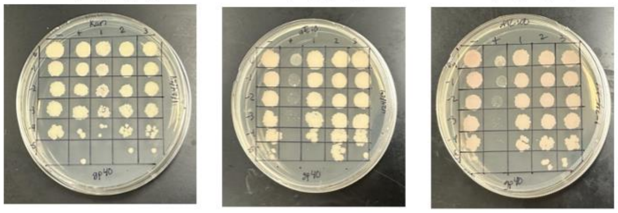

0 ng/ml aTc | 10 ng/ml aTc | 100 ng/ml aTc

Lane	Gene ID	Plasmid name	Gene name	Toxic/Non-toxic	Colony color on 100 ng/ml aTc plate*
- Non-toxic control	--	pExTra03	Fruitloop *52* mutant	Non-toxic	++
+ Toxic control	--	pExTra02	Fruitloop *52*	Toxic	-
1		pExTra-Lebron 40	Lebron *40* replicate 1	Non-toxic	+
2		pExTra-Lebron 40	Lebron *40* replicate 2	Non-toxic	+
3		pExTra-Lebron 40	Lebron *40* replicate 3	Non-toxic	+

*Key: NG (no growth) - (no pink color) +(faint pink color) ++(obvious pink color) +++ (dark pink color)

Images taken after 5 days at 37 ºC

Gene *41*; Score *0*

Dilution factor: 10^0, 10^{-1}, 10^{-2}, 10^{-3}, 10^{-4}, 10^{-5}

Lanes: - + 1 2 3 | - + 1 2 3 | - + 1 2 3

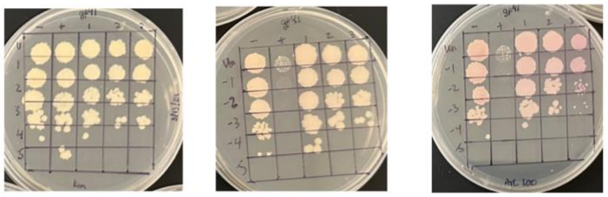

0 ng/ml aTc | 10 ng/ml aTc | 100 ng/ml aTc

Lane	Gene ID	Plasmid name	Gene name	Toxic/Non-toxic	Colony color on 100 ng/ml aTc plate*
- Non-toxic control	--	pExTra03	Fruitloop *52* mutant	Non-toxic	++
+ Toxic control	--	pExTra02	Fruitloop *52*	Toxic	-
1		pExTra-Lebron 41	Lebron *41* replicate 1	Non-toxic	++
2		pExTra-Lebron 41	Lebron *41* replicate 2	Non-toxic	++
3		pExTra-Lebron 41	Lebron *41* replicate 3	Non-toxic	++

*Key: NG (no growth) - (no pink color) +(faint pink color) ++(obvious pink color) +++ (dark pink color)

Images taken after 5 days at 37 ºC

Gene *42*; Score *0*

Dilution factor: 10^0, 10^{-1}, 10^{-2}, 10^{-3}, 10^{-4}, 10^{-5}

Lanes: - + 1 2 3 | - + 1 2 3 | - + 1 2 3

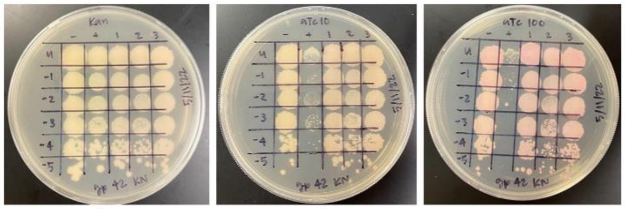

0 ng/ml aTc | 10 ng/ml aTc | 100 ng/ml aTc

Lane	Gene ID	Plasmid name	Gene name	Toxic/Non-toxic	Colony color on 100 ng/ml aTc plate*
- Non-toxic control	--	pExTra03	Fruitloop *52* mutant	Non-toxic	+
+ Toxic control	--	pExTra02	Fruitloop *52*	Toxic	-
1		pExTra-Lebron 42	Lebron 42 replicate 1	Non-toxic	++
2		pExTra-Lebron 42	Lebron 42 replicate 2	Non-toxic	++
3		pExTra-Lebron 42	Lebron 42 replicate 3	Non-toxic	++

*Key: NG (no growth) - (no pink color) +(faint pink color) ++(obvious pink color) +++ (dark pink color)

Images taken after 5 days at 37 ºC

Gene *43*; Score *0*

Dilution factor: 10^0, 10^{-1}, 10^{-2}, 10^{-3}, 10^{-4}, 10^{-5}

Lanes: - + 1 2 3 | - + 1 2 3 | - + 1 2 3

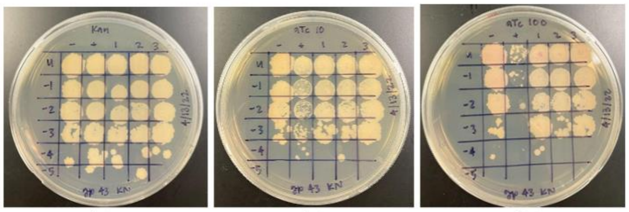

0 ng/ml aTc | 10 ng/ml aTc | 100 ng/ml aTc

Lane	Gene ID	Plasmid name	Gene name	Toxic/Non-toxic	Colony color on 100 ng/ml aTc plate*
- Non-toxic control	--	pExTra03	Fruitloop *52* mutant	Non-toxic	+
+ Toxic control	--	pExTra02	Fruitloop *52*	Toxic	-
1		pExTra-Lebron 43	Lebron 43 replicate 1	Non-toxic	+
2		pExTra-Lebron 43	Lebron 43 replicate 2	Non-toxic	+
3		pExTra-Lebron 43	Lebron 43 replicate 3	Non-toxic	+

*Key: NG (no growth) - (no pink color) +(faint pink color) ++(obvious pink color) +++ (dark pink color)

Images taken after 5 days at 37 ºC

Gene *44*; Score *0*

Dilution factor: 10^0, 10^{-1}, 10^{-2}, 10^{-3}, 10^{-4}, 10^{-5}

Lanes: - + 1 2 3 | - + 1 2 3 | - + 1 2 3

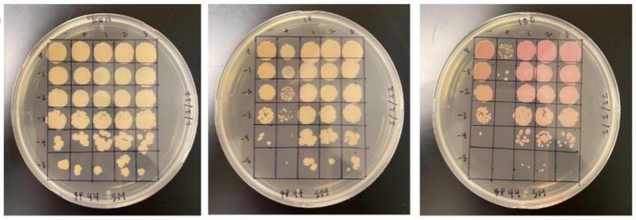

0 ng/ml aTc | 10 ng/ml aTc | 100 ng/ml aTc

Lane	Gene ID	Plasmid name	Gene name	Toxic/Non-toxic	Colony color on 100 ng/ml aTc plate*
+ Toxic control	--	pExTra02	Fruitloop *52*	Toxic	-
- Non-toxic control	--	pExTra03	Fruitloop *52* mutant	Non-toxic	+
1		pExTra-Lebron 44	Lebron *44* replicate 1	Non-toxic	++
2		pExTra-Lebron 44	Lebron *44* replicate 2	Non-toxic	++
3		pExTra-Lebron 44	Lebron *44* replicate 3	Non-toxic	++

*Key: NG (no growth) - (no pink color) +(faint pink color) ++(obvious pink color) +++ (dark pink color)

Images taken after 5 days at 37 ºC

Gene *45*; Score *0*

Dilution factor: 10^0, 10^{-1}, 10^{-2}, 10^{-3}, 10^{-4}, 10^{-5}

Lanes: - + 1 2 3 | - + 1 2 3 | - + 1 2 3

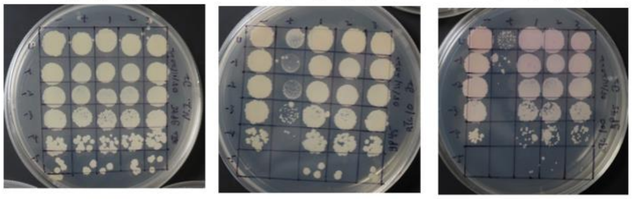

0 ng/ml aTc | 10 ng/ml aTc | 100 ng/ml aTc

Lane	Gene ID	Plasmid name	Gene name	Toxic/Non-toxic	Colony color on 100 ng/ml aTc plate*
- Non-toxic control	--	pExTra03	Fruitloop *52* mutant	Non-toxic	+
+ Toxic control	--	pExTra02	Fruitloop *52*	Non-Toxic	-
1		pExTra-Lebron 45	Lebron *45* replicate 1	Non-Toxic	++
2		pExTra-Lebron 45	Lebron *45* replicate 2	Non-Toxic	++
3		pExTra-Lebron 45	Lebron *45* replicate 3	Non-Toxic	++

*Key: NG (no growth) - (no pink color) +(faint pink color) ++(obvious pink color) +++ (dark pink color)

Images taken after 5 days at 37 ºC

Gene *47*; Score *2*

Dilution factor: 10^0, 10^{-1}, 10^{-2}, 10^{-3}, 10^{-4}, 10^{-5}

Lanes: - + 1 2 3 | - + 1 2 3 | - + 1 2 3

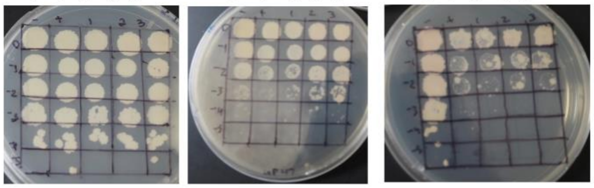

0 ng/ml aTc | 10 ng/ml aTc | 100 ng/ml aTc

Lane	Gene ID	Plasmid name	Gene name	Toxic/Non-toxic	Colony color on 100 ng/ml aTc plate*
+ Toxic control	--	pExTra02	Fruitloop *52*	Toxic	+
- Non-toxic control	--	pExTra03	Fruitloop *52* mutant	Non-toxic	-
1		pExTra-Lebron 47	Lebron *47* replicate 1	Toxic	+
2		pExTra-Lebron 47	Lebron *47* replicate 2	Toxic	+
3		pExTra-Lebron 47	Lebron *47* replicate 3	Toxic	+

*Key: NG (no growth) - (no pink color) +(faint pink color) ++(obvious pink color) +++ (dark pink color)

Images taken after 5 days at 37 ºC

Gene *48*; Score *0*

Dilution factor: 10^0, 10^{-1}, 10^{-2}, 10^{-3}, 10^{-4}, 10^{-5}

Lanes: - + 1 2 3 | - + 1 2 3 | - + 1 2 3

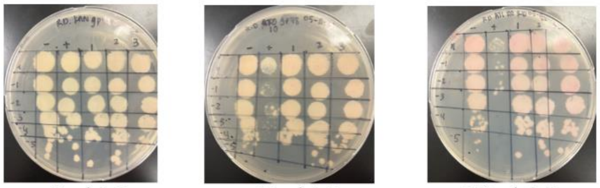

0 ng/ml aTc | 10 ng/ml aTc | 100 ng/ml aTc

Lane ll	Gene ID	Plasmid name	Gene name	Toxic/Non-toxic	Colony color on 100 ng/ml aTc plate*
- Non-toxic control	--	pExTra03	Fruitloop *52 mutant*	Non-toxic	+++
+ Toxic control	--	pExTra02	Fruitloop *52*	Toxic	+
1		pExTra-Lebron 48	Lebron 48 replicate 1	Non-toxic	++
2		pExTra-Lebron 48	Lebron 48 replicate 2	Non-toxic	++
3		pExTra-Lebron 48	Lebron 48 replicate 3	Non-toxic	++

*Key: NG (no growth) - (no pink color) +(faint pink color) ++(obvious pink color) +++ (dark pink color)

Images taken after 5 days at 37 ºC

Gene *49*; Score *1*

Dilution factor: 10^0, 10^{-1}, 10^{-2}, 10^{-3}, 10^{-4}, 10^{-5}

Lanes: - + 1 2 3 | - + 1 2 3 | - + 1 2 3

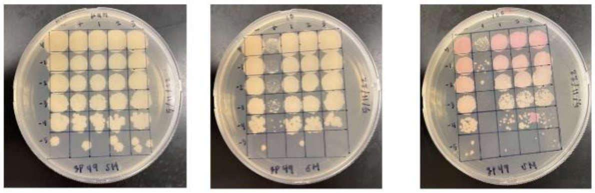

0 ng/ml aTc | 10 ng/ml aTc | 100 ng/ml aTc

Lane	Gene ID	Plasmid name	Gene name	Toxic/Non-toxic	Colony color on 100 ng/ml aTc plate*
+ Toxic control	--	pExTra02	Fruitloop *52*	Toxic	-
- Non-toxic control	--	pExTra03	Fruitloop *52* mutant	Non-toxic	++
1		pExTra-Lebron 49	Lebron *49* replicate 1	Toxic	++
2		pExTra-Lebron 49	Lebron *49* replicate 2	Toxic	++
3		pExTra-Lebron 49	Lebron *49* replicate 3	Toxic	++

*Key: NG (no growth) - (no pink color) +(faint pink color) ++(obvious pink color) +++ (dark pink color)

Gene *50*; Score *2*

Images taken after 5 days at 37 ºC

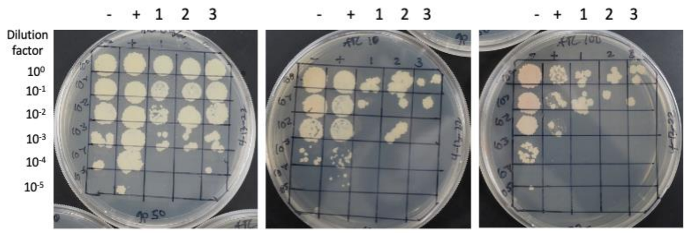

0 ng/ml aTc | 10 ng/ml aTc | 100 ng/ml aTc

Lane	Gene ID	Plasmid name	Gene name	Toxic/Non-toxic	Colony color on 100 ng/ml aTc plate*
+ Toxic control	--	pExTra02	Fruitloop *52*	Toxic	-
- Non-toxic control	--	pExTra03	Fruitloop *52* mutant	Non-toxic	++
1		pExTra-Lebron 50	Lebron 50 replicate 1	toxic	+
2		pExTra-Lebron 50	Lebron 50 replicate 2	toxic	+
3		pExTra-Lebron 50	Lebron 50 replicate 3	toxic	+

*Key: NG (no growth) - (no pink color) +(faint pink color) ++(obvious pink color) +++ (dark pink color)

Gene *51*; Score *0*

Images taken after 5 days at 37 ºC

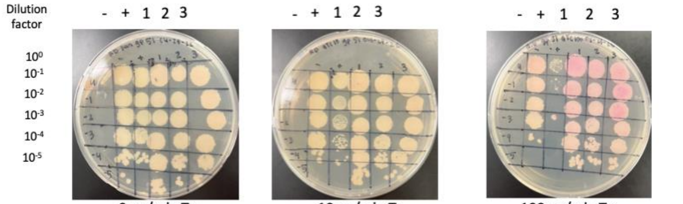

0 ng/ml aTc | 10 ng/ml aTc | 100 ng/ml aTc

Lane	Gene ID	Plasmid name	Gene name	Toxic/Non-toxic	Colony color on 100 ng/ml aTc plate*
- Non-toxic control	--	pExTra03	Fruitloop *52 mutant*	Non-toxic	++
+ Toxic control	--	pExTra02	Fruitloop *52*	Toxic	+
1		pExTra-Lebron 51	Lebron 51 replicate 1	Non-Toxic	+++
2		pExTra-Lebron 51	Lebron 51 replicate 2	Non-Toxic	+++
3		pExTra-Lebron 51	Lebron 51 replicate 3	Non-Toxic	+++

*Key: NG (no growth) - (no pink color) +(faint pink color) ++(obvious pink color) +++ (dark pink color)

Gene *52*; Score *0*

Images taken after 5 days at 37 ºC

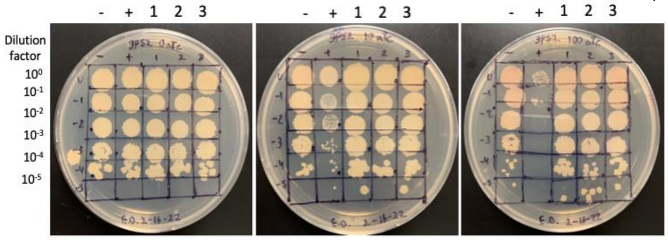

0 ng/ml aTc | 10 ng/ml aTc | 100 ng/ml aTc

Lane	Gene ID	Plasmid name	Gene name	Toxic/Non-toxic	Colony color on 100 ng/ml aTc plate*
+ Toxic control	--	pExTra02	Fruitloop *52*	Toxic	-
- Non-toxic control	--	pExTra03	Fruitloop *52* mutant	Non-toxic	++
1		pExTra-Lebron 52	Lebron 52 replicate 1	Non-toxic	++
2		pExTra-Lebron 52	Lebron 52 replicate 2	Non-toxic	++
3		pExTra-Lebron 52	Lebron 52replicate 3	Non-toxic	++

*Key: NG (no growth) - (no pink color) +(faint pink color) ++(obvious pink color) +++ (dark pink color)

Gene *53*; Score *2*

Images taken after 5 days at 37 ºC

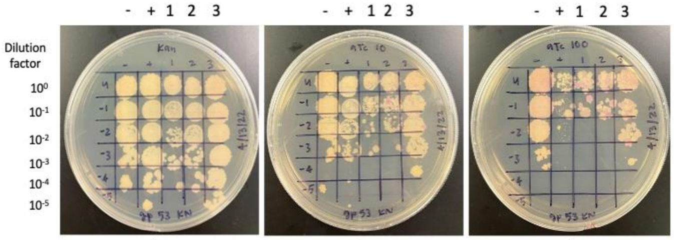

0 ng/ml aTc | 10 ng/ml aTc | 100 ng/ml aTc

Lane	Gene ID	Plasmid name	Gene name	Toxic/Non-toxic	Colony color on 100 ng/ml aTc plate*
- Non-toxic control	--	pExTra03	Fruitloop *52* mutant	Non-toxic	+
+ Toxic control	--	pExTra02	Fruitloop *52*	Toxic	-
1		pExTra-Lebron 53	Lebron 53 replicate 1	Toxic	+
2		pExTra-Lebron 53	Lebron 53 replicate 2	Toxic	+
3		pExTra-Lebron 53	Lebron 53 replicate 3	Toxic	+

*Key: NG (no growth) - (no pink color) +(faint pink color) ++(obvious pink color) +++ (dark pink color)

Gene *54*; Score *0*

Images taken after 5 days at 37 ºC

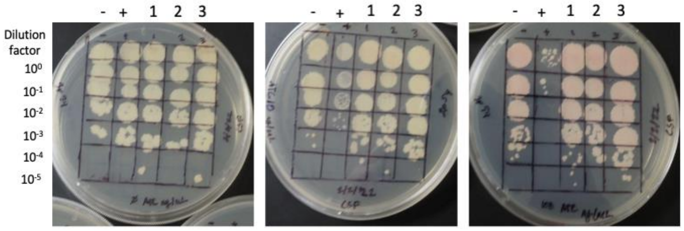

0 ng/ml aTc | 10 ng/ml aTc | 100 ng/ml aTc

Lane	Gene ID	Plasmid name	Gene name	Toxic/Non-toxic	Colony color on 100 ng/ml aTc plate*
- Non-toxic control	--	pExTra03	Fruitloop *52* mutant	Non-toxic	+
+ Toxic control	--	pExTra02	Fruitloop *52*	Toxic	-
1		pExTra-Lebron 54	Lebron *54* replicate 1	Non-toxic	++
2		pExTra-Lebron 54	Lebron *54* replicate 2	Non-toxic	++
3		pExTra-Lebron 54	Lebron *54* replicate 3	Non-toxic	++

*Key: NG (no growth) - (no pink color) +(faint pink color) ++(obvious pink color) +++ (dark pink color)

Gene *55*; Score *0*

Images taken after 5 days at 37 ºC

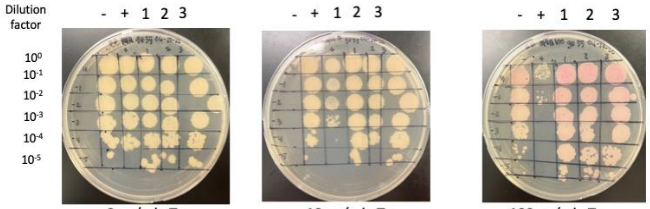

0 ng/ml aTc | 10 ng/ml aTc | 100 ng/ml aTc

Lane II	Gene ID	Plasmid name	Gene name	Toxic/Non-toxic	Colony color on 100 ng/ml aTc plate*
- Non-toxic control	--	pExTra03	Fruitloop *52 mutant*	Non-toxic	++
+ Toxic control	--	pExTra02	Fruitloop *52*	Toxic	+
1		pExTra-Lebron 55	Lebron 55 replicate 1	Non-Toxic	+++
2		pExTra-Lebron 55	Lebron 55 replicate 2	Non-Toxic	+++
3		pExTra-Lebron 55	Lebron 55 replicate 3	Non-Toxic	+++

*Key: NG (no growth) - (no pink color) +(faint pink color) ++(obvious pink color) +++ (dark pink color)

Gene *56*; Score *0*

Images taken after 5 days at 37 ºC

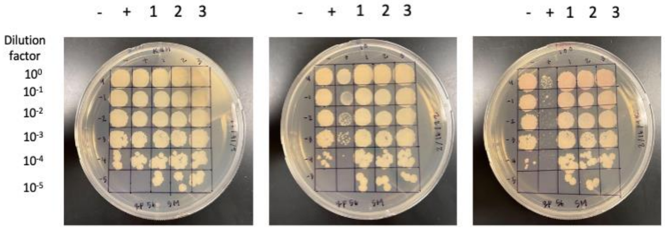

0 ng/ml aTc | 10 ng/ml aTc | 100 ng/ml aTc

Lane	Gene ID	Plasmid name	Gene name	Toxic/Non-toxic	Colony color on 100 ng/ml aTc plate*
+ Toxic control	--	pExTra02	Fruitloop *52*	Toxic	-
- Non-toxic control	--	pExTra03	Fruitloop *52* mutant	Non-toxic	+
1		pExTra-Lebron 56	Lebron *56* replicate 1	Non-toxic	+
2		pExTra-Lebron 56	Lebron *56* replicate 2	Non-toxic	+
3		pExTra-Lebron 56	Lebron 56 replicate 3	Non-toxic	+

*Key: NG (no growth) - (no pink color) +(faint pink color) ++(obvious pink color) +++ (dark pink color)

Gene *57*; Score *0*

Images taken after 5 days at 37 ºC

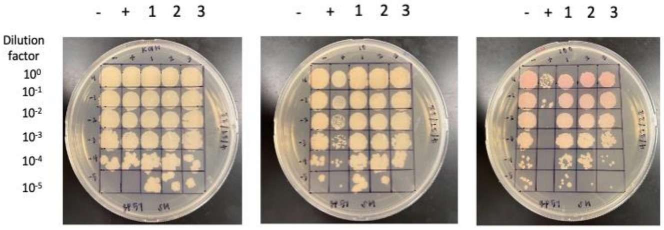

0 ng/ml aTc | 10 ng/ml aTc | 100 ng/ml aTc

Lane	Gene ID	Plasmid name	Gene name	Toxic/Non-toxic	Colony color on 100 ng/ml aTc plate*
+ Toxic control	--	pExTra02	Fruitloop *52*	Toxic	-
- Non-toxic control	--	pExTra03	Fruitloop *52* mutant	Non-toxic	++
1		pExTra-Lebron 57	Lebron *57* replicate 1	Non-toxic	++
2		pExTra-Lebron 57	Lebron *57* replicate 2	Non-toxic	++
3		pExTra-Lebron 57	Lebron *57* replicate 3	Non-toxic	++

*Key: NG (no growth) - (no pink color) +(faint pink color) ++(obvious pink color) +++ (dark pink color)

Images taken after 5 days at 37 ºC

Gene *58*; Score *0*

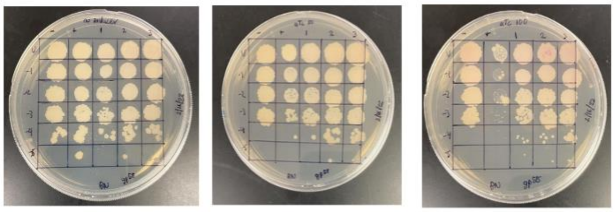

0 ng/ml aTc | 10 ng/ml aTc | 100 ng/ml aTc

Lane	Gene ID	Plasmid name	Gene name	Toxic/Non-toxic	Colony color on 100 ng/ml aTc plate*
- Non-toxic control	--	pExTra03	Fruitloop *52* mutant	Non-toxic	+
+ Toxic control	--	pExTra02	Fruitloop *52*	Toxic	-
1		pExTra-Lebron 58	Lebron *58* replicate 1	Non-toxic	+
2		pExTra-Lebron 58	Lebron *58* replicate 2	Non-toxic	+
3		pExTra-Lebron 58	Lebron *58* replicate 3	Non-toxic	+

*Key: NG (no growth) - (no pink color) +(faint pink color) ++(obvious pink color) +++ (dark pink color)

Images taken after 5 days at 37 ºC

Gene *59*; Score *3*

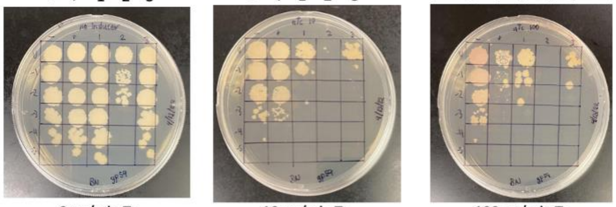

0 ng/ml aTc | 10 ng/ml aTc | 100 ng/ml aTc

Lane	Gene ID	Plasmid name	Gene name	Toxic/Non-toxic	Colony color on 100 ng/ml aTc plate*
+ Toxic control	--	pExTra02	Fruitloop *52*	Toxic	-
- Non-toxic control	--	pExTra03	Fruitloop *52* mutant	Non-toxic	+
1		pExTra-Lebron 59	Lebron *59* replicate 1	Toxic	-
2		pExTra-Lebron 59	Lebron *59* replicate 2	Toxic	NG
3		pExTra-Lebron 59	Lebron *59* replicate 3	Toxic	-

*Key: NG (no growth) - (no pink color) +(faint pink color) ++(obvious pink color) +++ (dark pink color)

Images taken after 5 days at 37 ºC

Gene *60*; Score *1*

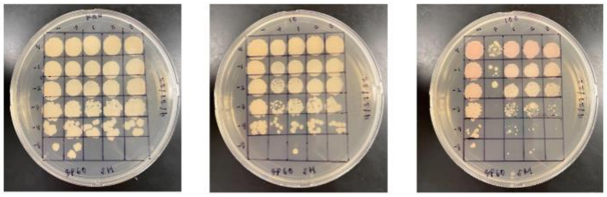

0 ng/ml aTc | 10 ng/ml aTc | 100 ng/ml aTc

Lane	Gene ID	Plasmid name	Gene name	Toxic/Non-toxic	Colony color on 100 ng/ml aTc plate*
+ Toxic control	--	pExTra02	Fruitloop *52*	Toxic	-
- Non-toxic control	--	pExTra03	Fruitloop *52* mutant	Non-toxic	+
1		pExTra-Lebron 60	Lebron *60* replicate 1	Toxic	+
2		pExTra-Lebron 60	Lebron *60* replicate 2	Toxic	+
3		pExTra-Lebron 60	Lebron *60* replicate 3	Toxic	+

*Key: NG (no growth) - (no pink color) +(faint pink color) ++(obvious pink color) +++ (dark pink color)

Images taken after 5 days at 37 ºC

Gene *61*; Score *0*

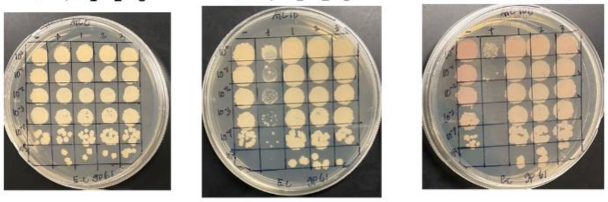

0 ng/ml aTc | 10 ng/ml aTc | 100 ng/ml aTc

Lane	Gene ID	Plasmid name	Gene name	Toxic/Non-toxic	Colony color on 100 ng/ml aTc plate*
+ Toxic control	--	pExTra02	Fruitloop *52*	Toxic	-
- Non-toxic control	--	pExTra03	Fruitloop *52* mutant	Non-toxic	+
1		pExTra-Lebron 61	Lebron 61 replicate 1	Non-toxic	+
2		pExTra-Lebron 61	Lebron 61 replicate 2	Non-toxic	+
3		pExTra-Lebron 61	Lebron 61 replicate 3	Non-toxic	+

*Key: NG (no growth) - (no pink color) +(faint pink color) ++(obvious pink color) +++ (dark pink color)

Images taken after 5 days at 37 ºC

Gene *62*; Score *0*

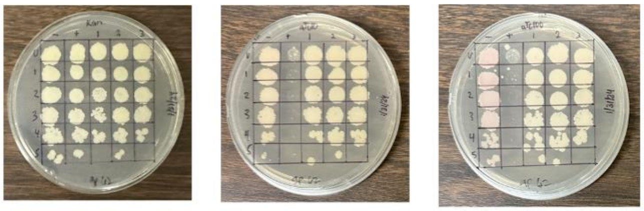

0 ng/ml aTc | 10 ng/ml aTc | 100 ng/ml aTc

Lane	Gene ID	Plasmid name	Gene name	Toxic/Non-toxic	Colony color on 100 ng/ml aTc plate*
- Non-toxic control	--	pExTra03	Fruitloop *52* mutant	Non-toxic	+
+ Toxic control	--	pExTra02	Fruitloop *52*	Toxic	-
1		pExTra-Lebron 62	Lebron *62* replicate 1	Non-toxic	-
2		pExTra-Lebron 62	Lebron *62* replicate 2	Non-toxic	-
3		pExTra-Lebron 62	Lebron *62* replicate 3	Non-toxic	-

*Key: NG (no growth) - (no pink color) +(faint pink color) ++(obvious pink color) +++ (dark pink color)

Images taken after 5 days at 37 ºC

Gene *63*; Score *0*

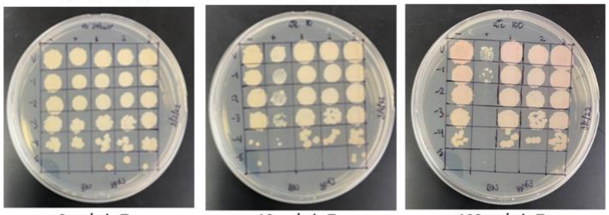

0 ng/ml aTc | 10 ng/ml aTc | 100 ng/ml aTc

Lane	Gene ID	Plasmid name	Gene name	Toxic/Non-toxic	Colony color on 100 ng/ml aTc plate*
- Non-toxic control	--	pExTra03	Fruitloop *52* mutant	Non-toxic	+
+ Toxic control	--	pExTra02	Fruitloop *52*	Toxic	-
1		pExTra-Lebron 63	Lebron *63* replicate 1	Non-toxic	++
2		pExTra-Lebron 63	Lebron *63* replicate 2	Non-toxic	++
3		pExTra-Lebron 63	Lebron *63* replicate 3	Non-toxic	++

*Key: NG (no growth) - (no pink color) +(faint pink color) ++(obvious pink color) +++ (dark pink color)

Images taken after 5 days at 37 ºC

Gene *64*; Score *1*

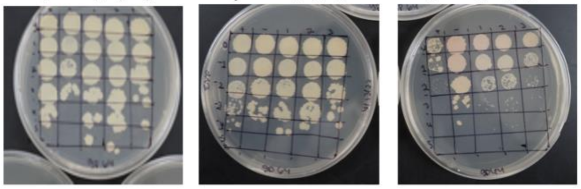

0 ng/ml aTc | 10 ng/ml aTc | 100 ng/ml aTc

Lane	Gene ID	Plasmid name	Gene name	Toxic/Non-toxic	Colony color on 100 ng/ml aTc plate*
+ Toxic control	--	pExTra02	Fruitloop *52*	Toxic	-
- Non-toxic control	--	pExTra03	Fruitloop *52* mutant	Non-toxic	++
1		pExTra-Lebron 64	Lebron *64* replicate 1	Toxic	+
2		pExTra-Lebron 64	Lebron *64* replicate 2	Toxic	+
3		pExTra-Lebron 64	Lebron *64* replicate 3	Toxic	+

*Key: NG (no growth) - (no pink color) +(faint pink color) ++(obvious pink color) +++ (dark pink color)

Images taken after 5 days at 37 ºC

Gene *65*; Score *0*

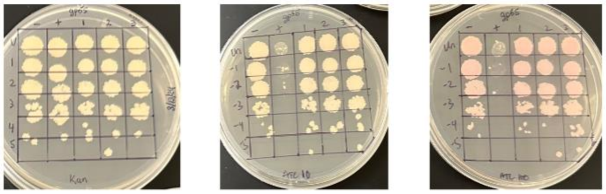

0 ng/ml aTc | 10 ng/ml aTc | 100 ng/ml aTc

Lane	Gene ID	Plasmid name	Gene name	Toxic/Non-toxic	Colony color on 100 ng/ml aTc plate*
- Non-toxic control	--	pExTra03	Fruitloop *52* mutant	Non-toxic	++
+ Toxic control	--	pExTra02	Fruitloop *52*	Toxic	-
1		pExTra-Lebron 65	Lebron *65* replicate 1	Non-toxic	++
2		pExTra-Lebron 65	Lebron *65* replicate 2	Non-toxic	++
3		pExTra-Lebron 65	Lebron *65* replicate 3	Non-toxic	++

*Key: NG (no growth) - (no pink color) +(faint pink color) ++(obvious pink color) +++ (dark pink color)

Gene *66*; Score *1*

Images taken after 5 days at 37 ºC

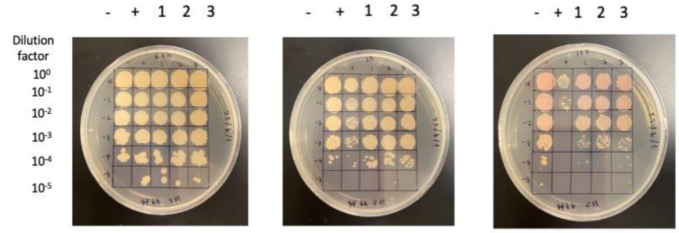

0 ng/ml aTc   10 ng/ml aTc   100 ng/ml aTc

Lane	Gene ID	Plasmid name	Gene name	Toxic/Non-toxic	Colony color on 100 ng/ml aTc plate*
+ Toxic control	--	pExTra02	Fruitloop *52*	Toxic	-
- Non-toxic control	--	pExTra03	Fruitloop *52* mutant	Non-toxic	++
1		pExTra-Lebron 66	Lebron *66* replicate 1	Toxic	++
2		pExTra-Lebron 66	Lebron *66* replicate 2	Toxic	++
3		pExTra-Lebron 66	Lebron 66 replicate 3	Toxic	++

*Key: NG (no growth) - (no pink color) +(faint pink color) ++(obvious pink color) +++ (dark pink color)

Gene *67*; Score *1*

Images taken after 5 days at 37 ºC

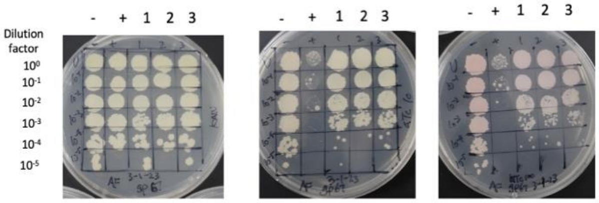

0 ng/ml aTc   10 ng/ml aTc   100 ng/ml aTc

Lane	Gene ID	Plasmid name	Gene name	Toxic/Non-toxic	Colony color on 100 ng/ml aTc plate*
+ Toxic control	--	pExTra02	Fruitloop *52*	Toxic	-
- Non-toxic control	--	pExTra03	Fruitloop *52* mutant	Non-toxic	++
1		pExTra-Lebron *67*	Lebron *67* replicate 1	Toxic	+
2		pExTra-Lebron *67*	Lebron *67* replicate 2	Toxic	+
3		pExTra-Lebron *67*	Lebron *67* replicate 3	Toxic	+

*Key: NG (no growth) - (no pink color) +(faint pink color) ++(obvious pink color) +++ (dark pink color)

Gene *68*; Score *1*

Images taken after 5 days at 37 ºC

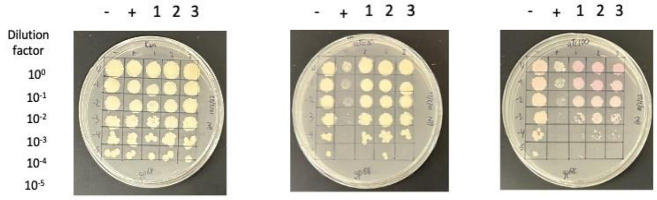

0 ng/ml aTc   10 ng/ml aTc   100 ng/ml aTc

Lane	Gene ID	Plasmid name	Gene name	Toxic/Non-toxic	Colony color on 100 ng/ml aTc plate*
- Non-toxic control	--	pExTra03	Fruitloop *52* mutant	Non-toxic	+
+ Toxic control	--	pExTra02	Fruitloop *52*	Toxic	-
1		pExTra-Lebron 68	Lebron *68* replicate 1	Toxic	++
2		pExTra-Lebron 68	Lebron *68* replicate 2	Toxic	++
3		pExTra-Lebron 68	Lebron *68* replicate 3	Toxic	++

*Key: NG (no growth) - (no pink color) +(faint pink color) ++(obvious pink color) +++ (dark pink color)

Gene *69*; Score *0*

Images taken after 5 days at 37 ºC

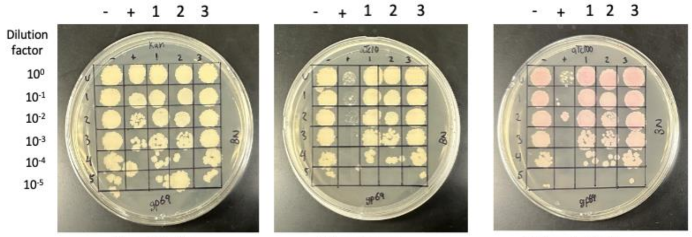

0 ng/ml aTc   10 ng/ml aTc   100 ng/ml aTc

Lane	Gene ID	Plasmid name	Gene name	Toxic/Non-toxic	Colony color on 100 ng/ml aTc plate*
- Non-toxic control	--	pExTra03	Fruitloop *52* mutant	Non-toxic	++
+ Toxic control	--	pExTra02	Fruitloop *52*	Toxic	++
1		pExTra-Lebron 69	Lebron *69* replicate 1	Non-toxic	++
2		pExTra-Lebron 69	Lebron *69* replicate 2	Non-toxic	++
3		pExTra-Lebron 69	Lebron *69* replicate 3	Non-toxic	++

*Key: NG (no growth) - (no pink color) +(faint pink color) ++(obvious pink color) +++ (dark pink color)

Gene *70*; Score *2*

Images taken after 5 days at 37 ºC

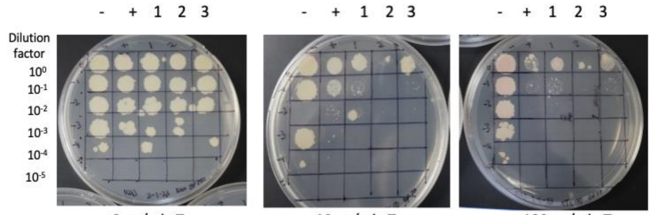

0 ng/ml aTc   10 ng/ml aTc   100 ng/ml aTc

Lane	Gene ID	Plasmid name	Gene name	Toxic/Non-toxic	Colony color on 100 ng/ml aTc plate*
+ Toxic control	--	pExTra02	Fruitloop *52*	Toxic	-
- Non-toxic control	--	pExTra03	Fruitloop *52* mutant	Non-toxic	+
1		pExTra-Lebron *70*	Lebron *70* replicate 1	Toxic	+
2		pExTra-Lebron *70*	Lebron *70* replicate 2	Toxic	-
3		pExTra-Lebron *70*	Lebron *70* replicate 3	Toxic	-

*Key: NG (no growth) - (no pink color) +(faint pink color) ++(obvious pink color) +++ (dark pink color)

Gene *71*; Score *3*

Images taken after 5 days at 37 ºC

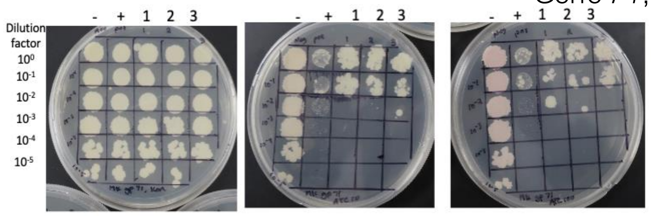

0 ng/ml aTc   10 ng/ml aTc   100 ng/ml aTc

Lane	Gene ID	Plasmid name	Gene name	Toxic/Non-toxic	Colony color on 100 ng/ml aTc plate*
+ Toxic control	--	pExTra02	Fruitloop 52	Toxic	-
- Non-toxic control	--	pExTra03	Fruitloop 52 mutant	Non-toxic	+
1		pExTra-Lebron 71	Lebron *71* replicate 1	Toxic	-
2		pExTra-Lebron 71	Lebron *71* replicate 2	Toxic	-
3		pExTra-Lebron 71	Lebron *71* replicate 3	Toxic	-

*Key: NG (no growth) - (no pink color) +(faint pink color) ++(obvious pink color) +++ (dark pink color)

Gene *72*; Score *0*

Images taken after 5 days at 37 ºC

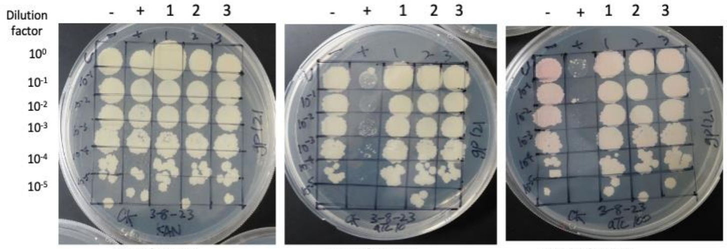

0 ng/ml aTc   10 ng/ml aTc   100 ng/ml aTc

Lane	Gene ID	Plasmid name	Gene name	Toxic/Non-toxic	Colony color on 100 ng/ml aTc plate*
- Non-toxic control	--	pExTra03	Fruitloop *52* mutant	Non-toxic	++
+ Toxic control	--	pExTra02	Fruitloop *52*	Toxic	–
1	–	pExTra-Lebron 72	Lebron *72* replicate 1	Non-toxic	–
2	–	pExTra-Lebron 72	Lebron *72* replicate 2	Non-toxic	–
3	–	pExTra-Lebron 72	Lebron *72* replicate 3	Non-toxic	–

*Key: NG (no growth) - (no pink color) +(faint pink color) ++(obvious pink color) +++ (dark pink color)

Gene *73*; Score *2*

Images taken after 5 days at 37 ºC

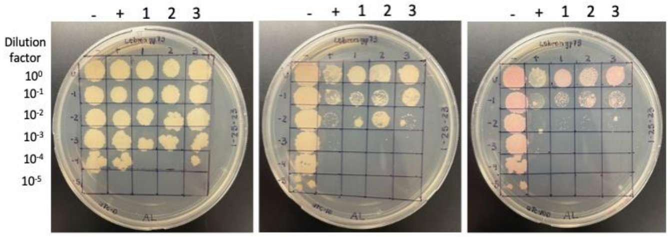

0 ng/ml aTc   10 ng/ml aTc   100 ng/ml aTc

Lane	Gene ID	Plasmid name	Gene name	Toxic/Non-toxic	Colony color on 100 ng/ml aTc plate*
+ Toxic control	--	pExTra02	Fruitloop *52*	Toxic	-
- Non-toxic control	--	pExTra03	Fruitloop *52* mutant	Non-toxic	+
1		pExTra-Lebron73	Lebron 73 replicate 1	Toxic	++
2		pExTra-Lebron73	Lebron 73 replicate 2	Toxic	++
3		pExTra-Lebron73	Lebron 73 replicate 3	Toxic	++

*Key: NG (no growth) - (no pink color) +(faint pink color) ++(obvious pink color) +++ (dark pink color)

Gene *74*; Score *1*

Images taken after 5 days at 37 ºC

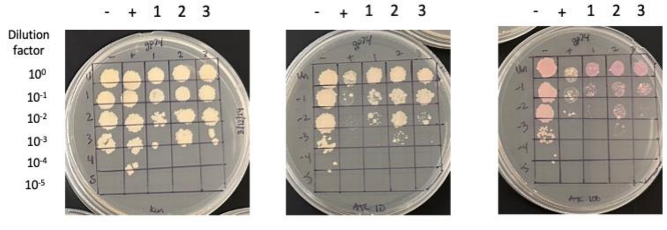

Lane	Gene ID	Plasmid name	Gene name	Toxic/Non-toxic	Colony color on 100 ng/ml aTc plate*
- Non-toxic control	--	pExTra03	Fruitloop *52* mutant	Non-toxic	++
+ Toxic control	--	pExTra02	Fruitloop *52*	Toxic	-
1		pExTra-Lebron 74	Lebron *74* replicate 1	Toxic	+++
2		pExTra-Lebron 74	Lebron *74* replicate 2	Toxic	+++
3		pExTra-Lebron 74	Lebron *74* replicate 3	Toxic	+++

*Key: NG (no growth) - (no pink color) +(faint pink color) ++(obvious pink color) +++ (dark pink color)

Gene *75*; Score *0*

Images taken after 5 days at 37 ºC

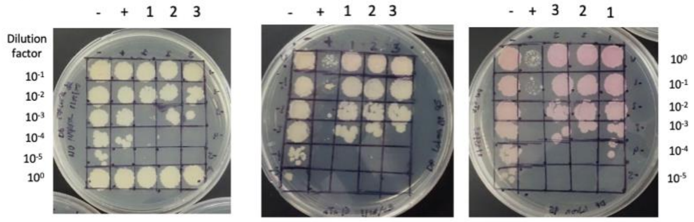

Lane	Gene ID	Plasmid name	Gene name	Toxic/Non-toxic	Colony color on 100 ng/ml aTc plate*
+ Toxic control	--	pExTra02	Fruitloop *52*	Toxic	-
- Non-toxic control	--	pExTra03	Fruitloop *52* mutant	Non-toxic	++
1		pExTra-Lebron 75	Lebron *75* replicate 1	Non-toxic	+++
2		pExTra-Lebron 75	Lebron *75* replicate 2	Non-toxic	+++
3		pExTra-Lebron 75	Lebron *75* replicate 3	Non-toxic	+++

*Key: NG (no growth) - (no pink color) +(faint pink color) ++(obvious pink color) +++ (dark pink color)

Gene *76*; Score *3*

Images taken after 5 days at 37 ºC

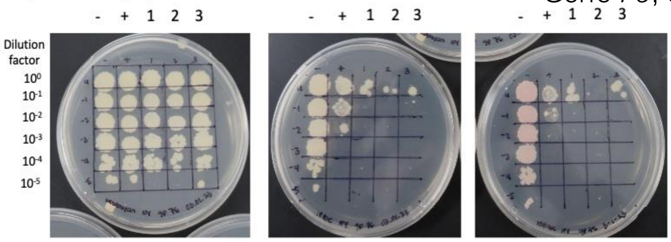

Lane	Gene ID	Plasmid name	Gene name	Toxic/Non-toxic	Colony color on 100 ng/ml aTc plate*
+ Toxic control	--	pExTra02	Fruitloop *52*	Toxic	-
- Non-toxic control	--	pExTra03	Fruitloop *52* mutant	Non-toxic	+
1	131436	pExTra-Lebron 76	Lebron 76 replicate 1	Toxic	-
2	131436	pExTra-Lebron 76	Lebron 76 replicate 2	Toxic	-
3	131436	pExTra-Lebron 76	Lebron 76 replicate 3	Toxic	-

*Key: NG (no growth) - (no pink color) +(faint pink color) ++(obvious pink color) +++ (dark pink color)

Gene *77*; Score *0*

Images taken after 5 days at 37 ºC

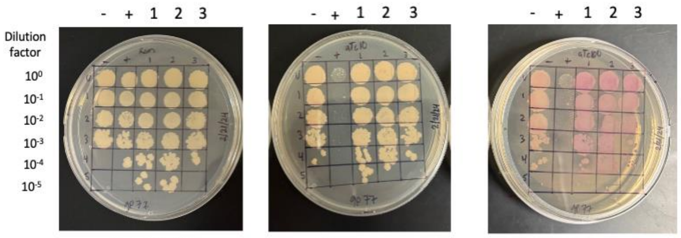

Lane	Gene ID	Plasmid name	Gene name	Toxic/Non-toxic	Colony color on 100 ng/ml aTc plate*
- Non-toxic control	--	pExTra03	Fruitloop *52* mutant	Non-toxic	++
+ Toxic control	--	pExTra02	Fruitloop *52*	Toxic	-
1		pExTra-Lebron 77	Lebron *77* replicate 1	Non-toxic	+++
2		pExTra-Lebron 77	Lebron *77* replicate 2	Non-toxic	+++
3		pExTra-Lebron 77	Lebron *77* replicate 3	Non-toxic	+++

*Key: NG (no growth) - (no pink color) +(faint pink color) ++(obvious pink color) +++ (dark pink color)

Gene *78*; Score *3*

Images taken after 5 days at 37 ºC

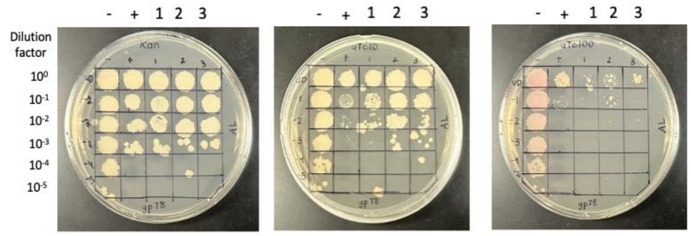

Lane	Gene ID	Plasmid name	Gene name	Toxic/Non-toxic	Colony color on 100 ng/ml aTc plate*
- Non-toxic control	--	pExTra03	Fruitloop *52* mutant	Non-toxic	++
+ Toxic control	--	pExTra02	Fruitloop *52*	Toxic	-
1		pExTra-Lebron 78	Lebron *78* replicate 1	Toxic	-
2		pExTra-Lebron 78	Lebron *78* replicate 2	Toxic	-
3		pExTra-Lebron 78	Lebron *78* replicate 3	Toxic	-

*Key: NG (no growth) - (no pink color) +(faint pink color) ++(obvious pink color) +++ (dark pink color)

Gene *79*; Score *0*

Images taken after 5 days at 37 ºC

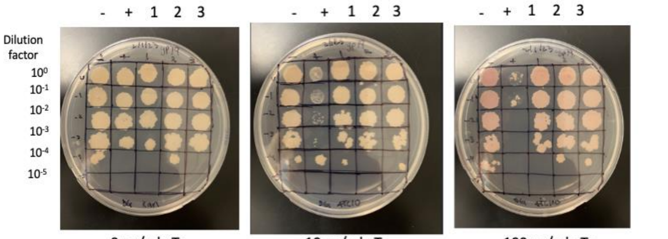

Lane	Gene ID	Plasmid name	Gene name	Toxic/Non-toxic	Colony color on 100 ng/ml aTc plate*
+ Toxic control	--	pExTra02	Fruitloop *52*	Toxic	-
- Non-toxic control	--	pExTra03	Fruitloop *52* mutant	Non-toxic	+++
1	131436	pExtra-Lebron 79	Lebron 79 replicate 1	Non-toxic	++
2	131436	pExTra-Lebron 79	Lebron 79 replicate 2	Non-toxic	++
3	131436	pExTra-Lebron 79	Lebron 79 replicate 3	Non-toxic	++

*Key: NG (no growth) - (no pink color) +(faint pink color) ++(obvious pink color) +++ (dark pink color)

Gene *80*; Score *1*

Images taken after 5 days at 37 ºC

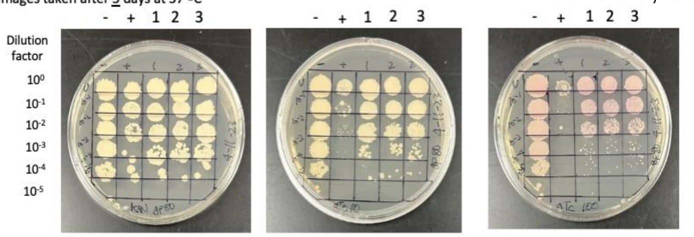

Lane	Gene ID	Plasmid name	Gene name	Toxic/Non-toxic	Colony color on 100 ng/ml aTc plate*
- Non-toxic control	--	pExTra03	Fruitloop *52* mutant	Non-toxic	++
+ Toxic control	--	pExTra02	Fruitloop *52*	Toxic	-
1		pExTra-Lebron 80	Lebron *80* replicate 1	Toxic	++
2		pExTra-Lebron 80	Lebron *80* replicate 2	Toxic	++
3		pExTra-Lebron 80	Lebron *80* replicate 3	Toxic	++

*Key: NG (no growth) - (no pink color) +(faint pink color) ++(obvious pink color) +++ (dark pink color)

Gene *81*; Score *3*

Images taken after 5 days at 37 ºC

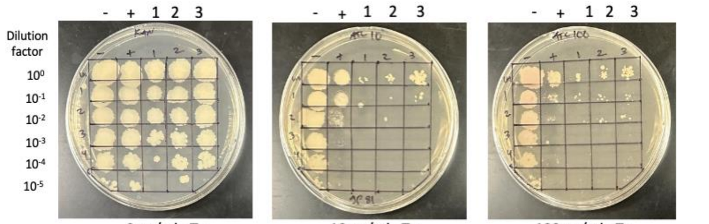

Lane	Gene ID	Plasmid name	Gene name	Toxic/Non-toxic	Colony color on 100 ng/ml aTc plate*
- Non-toxic control	--	pExTra03	Fruitloop 52 mutant	Non-toxic	+
+ Toxic control	--	pExTra02	Fruitloop *52*	Toxic	-
1		pExTra-Lebron 81	Lebron *81* replicate 1	Toxic	-
2		pExTra-Lebron 81	Lebron *81* replicate 2	Toxic	-
3		pExTra-Lebron 81	Lebron *81* replicate 3	Toxic	-

*Key: NG (no growth) - (no pink color) +(faint pink color) ++(obvious pink color) +++ (dark pink color)

Gene *82*; Score *0*

Images taken after 5 days at 37 ºC

Dilution factor: 10^0, 10^{-1}, 10^{-2}, 10^{-3}, 10^{-4}, 10^{-5}

Lanes: - + 1 2 3 (each plate)

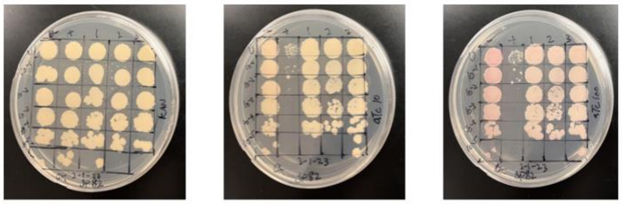

0 ng/ml aTc | 10 ng/ml aTc | 100 ng/ml aTc

Lane	Gene ID	Plasmid name	Gene name	Toxic/Non-toxic	Colony color on 100 ng/ml aTc plate*
- Non-toxic control	--	pExTra03	Fruitloop *52* mutant	Non-toxic	++
+ Toxic control	--	pExTra02	Fruitloop *52*	Toxic	–
1	–	pExTra-Lebron 82	Lebron *82* replicate 1	Non-toxic	+
2	–	pExTra-Lebron 82	Lebron *82* replicate 2	Non-toxic	+
3	–	pExTra-Lebron 82	Lebron *82* replicate 3	Non-toxic	+

*Key: NG (no growth) - (no pink color) +(faint pink color) ++(obvious pink color) +++ (dark pink color)

Gene *83*; Score *0*

Images taken after 5 days at 37 ºC

Dilution factor: 10^0, 10^{-1}, 10^{-2}, 10^{-3}, 10^{-4}, 10^{-5}

Lanes: - + 1 2 3 (each plate)

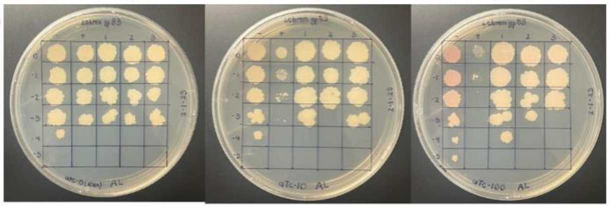

0 ng/ml aTc | 10 ng/ml aTc | 100 ng/ml aTc

Lane	Gene ID	Plasmid name	Gene name	Toxic/Non-toxic	Colony color on 100 ng/ml aTc plate*
+ Toxic control	--	pExTra02	Fruitloop *52*	Toxic	-
- Non-toxic control	--	pExTra03	Fruitloop *52* mutant	Non-toxic	+
1		pExTra-Lebron83	Lebron 83 replicate 1	Non-toxic	-
2		pExTra-Lebron83	Lebron 83 replicate 2	Non-toxic	-
3		pExTra-Lebron83	Lebron 83 replicate 3	Non-toxic	-

*Key: NG (no growth) - (no pink color) +(faint pink color) ++(obvious pink color) +++ (dark pink color)

Gene *84*; Score *0*

Images taken after 5 days at 37 ºC

Dilution factor: 10^0, 10^{-1}, 10^{-2}, 10^{-3}, 10^{-4}, 10^{-5}

Lanes: - + 1 2 3 (each plate)

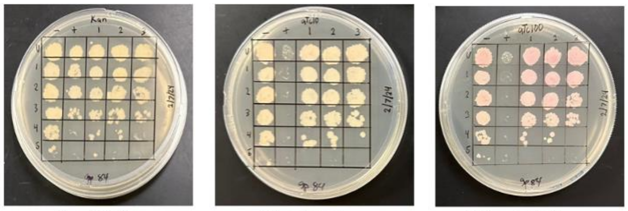

0 ng/ml aTc | 10 ng/ml aTc | 100 ng/ml aTc

Lane	Gene ID	Plasmid name	Gene name	Toxic/Non-toxic	Colony color on 100 ng/ml aTc plate*
- Non-toxic control	--	pExTra03	Fruitloop *52* mutant	Non-toxic	++
+ Toxic control	--	pExTra02	Fruitloop *52*	Toxic	-
1		pExTra-Lebron 84	Lebron *84* replicate 1	Non-toxic	++
2		pExTra-Lebron 84	Lebron *84* replicate 2	Non-toxic	++
3		pExTra-Lebron 84	Lebron *84* replicate 3	Non-toxic	++

*Key: NG (no growth) - (no pink color) +(faint pink color) ++(obvious pink color) +++ (dark pink color)

Gene *85*; Score *3*

Images taken after 5 days at 37 ºC

Dilution factor: 10^0, 10^{-1}, 10^{-2}, 10^{-3}, 10^{-4}, 10^{-5}

Lanes: - + 1 2 3 (each plate)

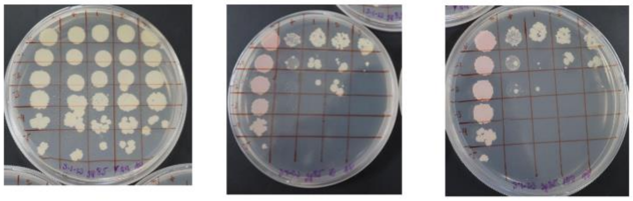

0 ng/ml aTc | 10 ng/ml aTc | 100 ng/ml aTc

Lane	Gene ID	Plasmid name	Gene name	Toxic/Non-toxic	Colony color on 100 ng/ml aTc plate*
+ Toxic control	--	pExTra02	Fruitloop *52*	Toxic	-
- Non-toxic control	--	pExTra03	Fruitloop *52* mutant	Non-toxic	++
1		pExTra-Lebron 85	Lebron *85* replicate 1	Toxic	-
2		pExTra-Lebron 85	Lebron *85* replicate 2	Toxic	-
3		pExTra-Lebron 85	Lebron *85* replicate 3	Toxic	-

*Key: NG (no growth) - (no pink color) +(faint pink color) ++(obvious pink color) +++ (dark pink color)

Gene *86*; Score *1*

Images taken after 5 days at 37 ºC

Dilution factor: 10^0, 10^{-1}, 10^{-2}, 10^{-3}, 10^{-4}, 10^{-5}

Lanes: - + 1 2 3 (each plate)

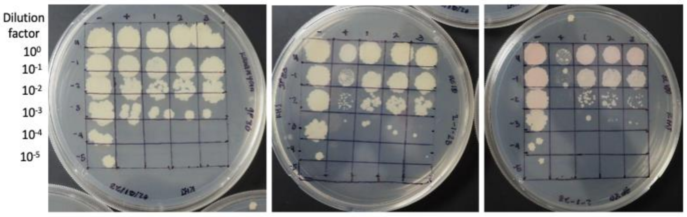

0 ng/ml aTc | 10 ng/ml aTc | 100 ng/ml aTc

Lane	Gene ID	Plasmid name	Gene name	Toxic/Non-toxic	Colony color on 100 ng/ml aTc plate*
+ Toxic control	--	pExTra02	Fruitloop *52*	Toxic	-
- Non-toxic control	--	pExTra03	Fruitloop *52* mutant	Non-toxic	+
1		pExTra-Lebron 86	Lebron 86 replicate 1	Toxic	+
2		pExTra-Lebron 86	Lebron 86 replicate 2	Toxic	+
3		pExTra-Lebron 86	Lebron 86 replicate 3	Toxic	+

*Key: NG (no growth) - (no pink color) +(faint pink color) ++(obvious pink color) +++ (dark pink color)

Gene *87*; Score *0*

Images taken after 5 days at 37 ºC

Dilution factor: 10^0, 10^{-1}, 10^{-2}, 10^{-3}, 10^{-4}, 10^{-5}

Lanes: - + 1 2 3 (each plate)

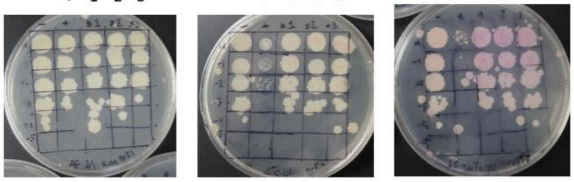

0 ng/ml aTc | 10 ng/ml aTc | 100 ng/ml aTc

Lane	Gene ID	Plasmid name	Gene name	Toxic/Non-toxic	Colony color on 100 ng/ml aTc plate*
+ Toxic control	--	pExTra02	Fruitloop *52*	Toxic	-
- Non-toxic control	--	pExTra03	Fruitloop *52* mutant	Non-toxic	+
1		pExTra-Lebron *87*	Lebron *87* replicate 1	Non-toxic	++
2		pExTra-Lebron *87*	Lebron *87* replicate 2	Non-toxic	++
3		pExTra-Lebron *87*	Lebron *87* replicate 3	Non-toxic	++

*Key: NG (no growth) - (no pink color) +(faint pink color) ++(obvious pink color) +++ (dark pink color)

Gene *88*; Score *0*

Images taken after 5 days at 37 ºC

Dilution factor: 10^0, 10^{-1}, 10^{-2}, 10^{-3}, 10^{-4}, 10^{-5}

Lanes: - + 1 2 1 (each plate)

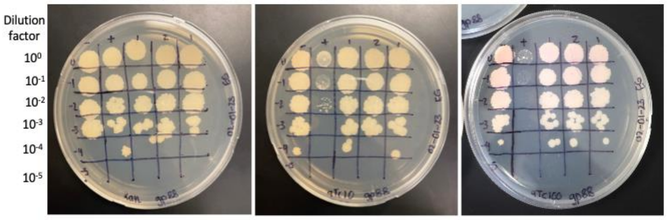

0 ng/ml aTc | 10 ng/ml aTc | 100 ng/ml aTc

Lane	Gene ID	Plasmid name	Gene name	Toxic/Non-toxic	Colony color on 100 ng/ml aTc plate*
+ Toxic control	--	pExTra02	Fruitloop *52*	Toxic	+
- Non-toxic control	--	pExTra03	Fruitloop *52* mutant	Non-toxic	-
1		pExTra-Lebron 88	Lebron 88 replicate 1	Non-toxic	+
2		pExTra-Lebron 88	Lebron 88 replicate 2	Non-toxic	+
3		pExTra-Lebron 88	Lebron 88 replicate 1	Non-toxic	+

*Key: NG (no growth) - (no pink color) +(faint pink color) ++(obvious pink color) +++ (dark pink color)

Gene *89*; Score *2*

Images taken after 5 days at 37 ºC

Dilution factor: 10^0, 10^{-1}, 10^{-2}, 10^{-3}, 10^{-4}, 10^{-5}

Lanes: - + 1 2 3 (each plate)

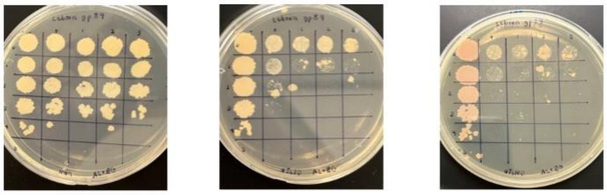

0 ng/ml aTc | 10 ng/ml aTc | 100 ng/ml aTc

Lane	Gene ID	Plasmid name	Gene name	Toxic/Non-toxic	Colony color on 100 ng/ml aTc plate*
- Non-toxic control	--	pExTra03	Fruitloop *52* mutant	Non-toxic	++
+ Toxic control	--	pExTra02	Fruitloop *52*	Toxic	-
1		pExTra-Lebron 89	Lebron *89* replicate 1	Toxic	+
2		pExTra-Lebron 89	Lebron *89* replicate 2	Toxic	+
3		pExTra-Lebron 89	Lebron *89* replicate 3	Toxic	+

*Key: NG (no growth) - (no pink color) +(faint pink color) ++(obvious pink color) +++ (dark pink color)

Images taken after 5 days at 37 ºC

Gene *90*; Score *0*

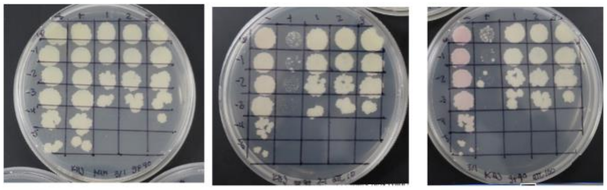

0 ng/ml aTc | 10 ng/ml aTc | 100 ng/ml aTc

Lane	Gene ID	Plasmid name	Gene name	Toxic/Non-toxic	Colony color on 100 ng/ml aTc plate*
+ Toxic control	--	pExTra02	Fruitloop *52*	Toxic	-
- Non-toxic control	--	pExTra03	Fruitloop *52* mutant	Non-toxic	++
1		pExTra-Lebron *90*	Lebron *90* replicate 1	Non-toxic	-
2		pExTra-Lebron *90*	Lebron *90* replicate 2	Non-toxic	-
3		pExTra-Lebron *90*	Lebron *90* replicate 3	Non-toxic	-

*Key: NG (no growth) - (no pink color) +(faint pink color) ++(obvious pink color) +++ (dark pink color)

Images taken after 5 days at 37 ºC

Gene *91* Score *0*

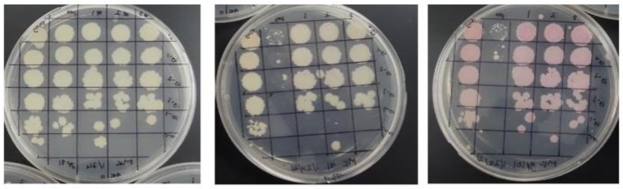

0 ng/ml aTc | 10 ng/ml aTc | 100 ng/ml aTc

Lane	Gene ID	Plasmid name	Gene name	Toxic/Non-toxic	Colony color on 100 ng/ml aTc plate*
+ Toxic control	--	pExTra02	Fruitloop 52	Toxic	-
- Non-toxic control	--	pExTra03	Fruitloop 52 mutant X	Non-toxic	++
1		pExTra-Lebron 91	Lebron *91* replicate 1	Non-toxic	++
2		pExTra-Lebron 91	Lebron *91* replicate 2	Non-toxic	++
3		pExTra-Lebron 91	Lebron *91* replicate 3	Non-toxic	++

*Key: NG (no growth) - (no pink color) +(faint pink color) ++(obvious pink color) +++ (dark pink color)

Images taken after 5 days at 37 ºC

Gene *92*; Score *0*

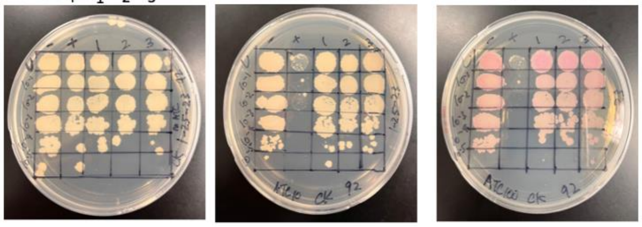

0 ng/ml aTc | 10 ng/ml aTc | 100 ng/ml aTc

Lane	Gene ID	Plasmid name	Gene name	Toxic/Non-toxic	Colony color on 100 ng/ml aTc plate*
- Non-toxic control	--	pExTra03	Fruitloop *52* mutant	Non-toxic	+
+ Toxic control	--	pExTra02	Fruitloop *52*	Toxic	-
1	–	pExTra-Lebron 92	Lebron *92* replicate 1	Non-toxic	++
2	–	pExTra-Lebron 92	Lebron *92* replicate 2	Non-toxic	++
3	–	pExTra-Lebron 92	Lebron *92* replicate 3	Non-toxic	++

*Key: NG (no growth) - (no pink color) +(faint pink color) ++(obvious pink color) +++ (dark pink color)

Images taken after 5 days at 37 ºC

Gene *93*; Score *3*

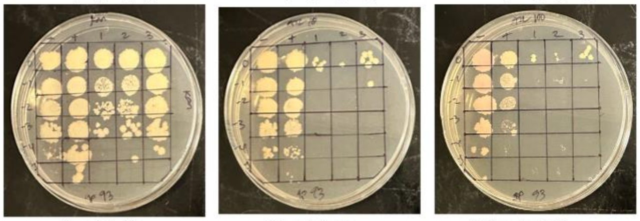

0 ng/ml aTc | 10 ng/ml aTc | 100 ng/ml aTc

Lane	Gene ID	Plasmid name	Gene name	Toxic/Non-toxic	Colony color on 100 ng/ml aTc plate*
- Non-toxic control	--	pExTra03	Fruitloop *52* mutant	Non-toxic	+
+ Toxic control	--	pExTra02	Fruitloop *52*	Toxic	-
1		pExTra-Lebron 93	Lebron *93* replicate 1	Toxic	+
2		pExTra-Lebron 93	Lebron *93* replicate 2	Toxic	+
3		pExTra-Lebron 93	Lebron *93* replicate 3	Toxic	+

*Key: NG (no growth) - (no pink color) +(faint pink color) ++(obvious pink color) +++ (dark pink color)

Images taken after 5 days at 37 ºC

Gene *94*; Score *0*

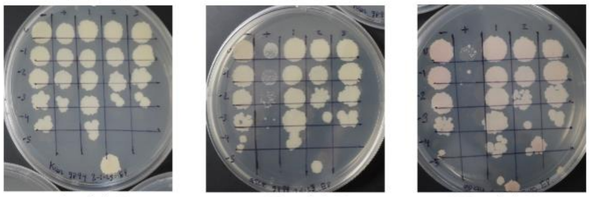

0 ng/ml aTc | 10 ng/ml aTc | 100 ng/ml aTc

Lane	Gene ID	Plasmid name	Gene name	Toxic/Non-toxic	Colony color on 100 ng/ml aTc plate*
- Non-toxic control	--	pExTra03	Fruitloop *52* mutant	Non-toxic	+
+ Toxic control	--	pExTra02	Fruitloop *52*	Toxic	-
1	–	pExTra-Lebron 94	Lebron *94* replicate 1	Non-toxic	+
2	–	pExTra-Lebron 94	Lebron *94* replicate 2	Non-toxic	+
3	–	pExTra-Lebron 94	Lebron *94* replicate 3	Non-toxic	+

*Key: NG (no growth) - (no pink color) +(faint pink color) ++(obvious pink color) +++ (dark pink color)

Images taken after 5 days at 37 ºC

Gene *95*; Score *0*

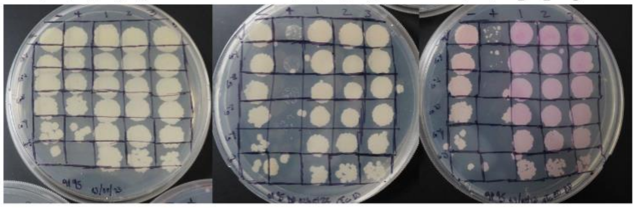

0 ng/ml aTc | 10 ng/ml aTc | 100 ng/ml aTc

Lane	Gene ID	Plasmid name	Gene name	Toxic/Non-toxic	Colony color on 100 ng/ml aTc plate*
+ Toxic control	--	pExTra02	Fruitloop *52*	Toxic	-
- Non-toxic control	--	pExTra03	Fruitloop *52* mutant	Non-toxic	++
1	--	pExTra-Lebron 95	Lebron *95* replicate 1	Non-toxic	+++
2	--	pExTra-Lebron 95	Lebron *95* replicate 2	Non-toxic	+++
3	--	pExTra-Lebron 95	Lebron *95* replicate 3	Non-toxic	+++

*Key: NG (no growth) - (no pink color) +(faint pink color) ++(obvious pink color) +++ (dark pink color)

Images taken after 5 days at 37 ºC

Gene *96*; Score *0*

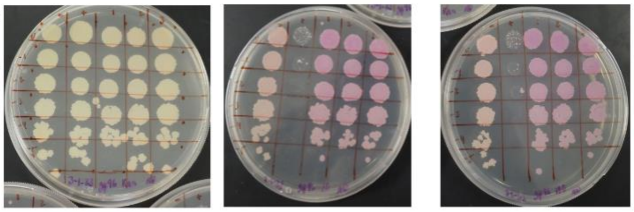

0 ng/ml aTc | 10 ng/ml aTc | 100 ng/ml aTc

Lane	Gene ID	Plasmid name	Gene name	Toxic/Non-toxic	Colony color on 100 ng/ml aTc plate*
+ Toxic control	--	pExTra02	Fruitloop *52*	Toxic	+
- Non-toxic control	--	pExTra03	Fruitloop *52* mutant	Non-toxic	-
1		pExTra-Lebron 96	Lebron 96 replicate 1	Non-toxic	+++
2		pExTra-Lebron 96	Lebron 96 replicate 2	Non-toxic	+++
3		pExTra-Lebron 96	Lebron 96 replicate 3	Non-toxic	+++

*Key: NG (no growth) - (no pink color) +(faint pink color) ++(obvious pink color) +++ (dark pink color)

Images taken after 5 days at 37 ºC

Gene *97*; Score *0*

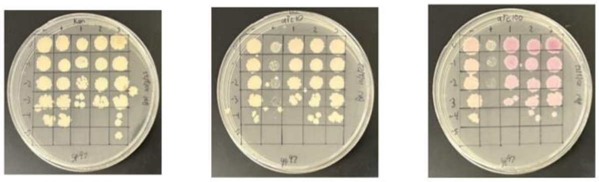

0 ng/ml aTc | 10 ng/ml aTc | 100 ng/ml aTc

Lane	Gene ID	Plasmid name	Gene name	Toxic/Non-toxic	Colony color on 100 ng/ml aTc plate*
- Non-toxic control	--	pExTra03	Fruitloop *52* mutant	Non-toxic	+
+ Toxic control	--	pExTra02	Fruitloop *52*	Toxic	-
1		pExTra-Lebron 97	Lebron *97* replicate 1	Non-toxic	++
2		pExTra-Lebron 97	Lebron *97* replicate 2	Non-toxic	++
3		pExTra-Lebron 97	Lebron *97* replicate 3	Non-toxic	+++

*Key: NG (no growth) - (no pink color) +(faint pink color) ++(obvious pink color) +++ (dark pink color)

Gene *98*; Score *0*

Images taken after 5 days at 37 ºC

- + 1 2 3   - + 1 2 3   - + 1 2 3

Dilution factor: 10^{0}, 10^{-1}, 10^{-2}, 10^{-3}, 10^{-4}, 10^{-5}

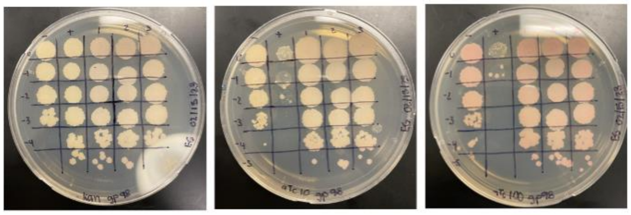

0 ng/ml aTc   10 ng/ml aTc   100 ng/ml aTc

Lane	Gene ID	Plasmid name	Gene name	Toxic/Non-toxic	Colony color on 100 ng/ml aTc plate*
+ Toxic control	--	pExTra02	Fruitloop *52*	Toxic	-
- Non-toxic control	--	pExTra03	Fruitloop *52* mutant	Non-toxic	+
1		pExTra-Lebron 98	Lebron 98 replicate 1	Non-Toxic	+
2		pExTra-Lebron 98	Lebron 98 replicate 2	Non-Toxic	+
3		pExTra-Lebron 98	Lebron *98* replicate 3	Non-Toxic	+

*Key: NG (no growth) - (no pink color) +(faint pink color) ++(obvious pink color) +++ (dark pink color)

Gene *99*; Score *3*

Images taken after 5 days at 37 ºC

- + 1 2 3   + - 1 2 3   - + 1 2 3

Dilution factor: 10^{0}, 10^{-1}, 10^{-2}, 10^{-3}, 10^{-4}, 10^{-5}

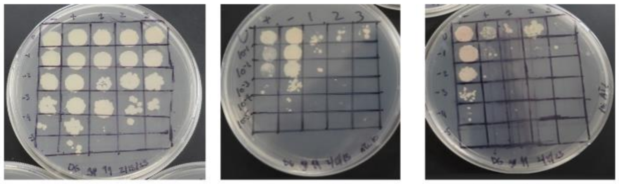

0 ng/ml aTc   10 ng/ml aTc   100 ng/ml aTc

Lane	Gene ID	Plasmid name	Gene name	Toxic/Non-toxic	Colony color on 100 ng/ml aTc plate*
+ Toxic control	--	pExTra02	Fruitloop *52*	Toxic	-
- Non-toxic control	--	pExTra03	Fruitloop *52* mutant	Non-toxic	+
1		pExtra-Lebron 99	Lebron 99 replicate 1	Toxic	-
2		pExTra-Lebron 99	Lebron 99 replicate 2	Toxic	-
3		pExTra-Lebron 99	Lebron 99 replicate 3	Toxic	NG

*Key: NG (no growth) - (no pink color) +(faint pink color) ++(obvious pink color) +++ (dark pink color)

Gene *100*; Score *1*

Images taken after 5 days at 37 ºC

- + 1 2 3   - + 1 2 3   - + 1 2 3

Dilution factor: 10^{0}, 10^{-1}, 10^{-2}, 10^{-3}, 10^{-4}, 10^{-5}

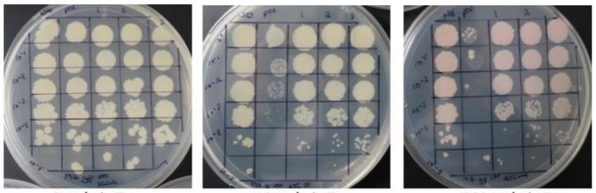

0 ng/ml aTc   10 ng/ml aTc   100 ng/ml aTc

Lane	Gene ID	Plasmid name	Gene name	Toxic/Non-toxic	Colony color on 100 ng/ml aTc plate*
+ Toxic control	--	pExTra02	Fruitloop *52*	Toxic	-
- Non-toxic control	--	pExTra03	Fruitloop *52* mutant	Non-toxic	+
1		pExTra-Lebron *100*	Lebron *100* replicate 1	Toxic	++
2		pExTra-Lebron *100*	Lebron *100* replicate 2	Toxic	++
3		pExTra-Lebron *100*	Lebron *100* replicate 3	Toxic	++

*Key: NG (no growth) - (no pink color) +(faint pink color) ++(obvious pink color) +++ (dark pink color)

Gene *101*; Score *0*

Images taken after 5 days at 37 ºC

- + 1 2 3   - + 1 2 3   - + 1 2 3

Dilution factor: 10^{0}, 10^{-1}, 10^{-2}, 10^{-3}, 10^{-4}, 10^{-5}

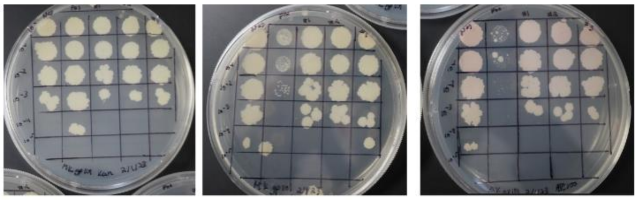

0 ng/ml aTc   10 ng/ml aTc   100 ng/ml aTc

Lane	Gene ID	Plasmid name	Gene name	Toxic/Non-toxic	Colony color on 100 ng/ml aTc plate*
+ Toxic control	--	pExTra02	Fruitloop 52	Toxic	-
- Non-toxic control	--	pExTra03	Fruitloop 52 mutant X	Non-toxic	+
1		pExTra-Lebron 101	Lebron *101* replicate 1	Non-toxic	+
2		pExTra-Lebron 101	Lebron *101* replicate 2	Non-toxic	+
3		pExTra-Lebron 101	Lebron *101* replicate 3	Non-toxic	+

*Key: NG (no growth) - (no pink color) +(faint pink color) ++(obvious pink color) +++ (dark pink color)

Gene 104; Score *0*

Images taken after 5 days at 37 ºC

- + 1 2 3   - + 1 2 3   - + 1 2 3

Dilution factor: 10^{0}, 10^{-1}, 10^{-2}, 10^{-3}, 10^{-4}, 10^{-5}

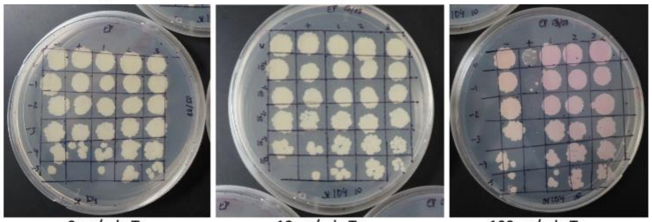

0 ng/ml aTc   10 ng/ml aTc   100 ng/ml aTc

Lane	Gene ID	Plasmid name	Gene name	Toxic/Non-toxic	Colony color on 100 ng/ml aTc plate*
- Non-toxic control	--	pExTra03	Fruitloop *52* mutant	Non-toxic	+
+ Toxic control	--	pExTra02	Fruitloop *52*	Toxic	-
1	–	pExTra-Lebron 104	Lebron *104* replicate 1	Non-toxic	++
2	–	pExTra-Lebron 104	Lebron *104* replicate 2	Non-toxic	++
3	–	pExTra-Lebron 104	Lebron *104* replicate 3	Non-toxic	++

*Key: NG (no growth) - (no pink color) +(faint pink color) ++(obvious pink color) +++ (dark pink color)

Gene *110*; Score *0*

Images taken after 5 days at 37 ºC

- + 1 2 3   - + 1 2 3   - + 1 2 3

Dilution factor: 10^{0}, 10^{-1}, 10^{-2}, 10^{-3}, 10^{-4}, 10^{-5}

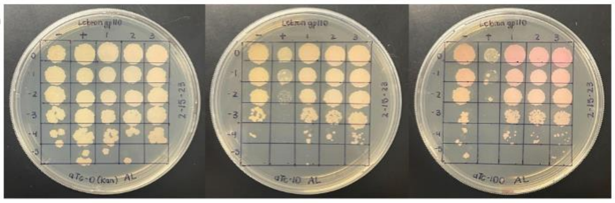

0 ng/ml aTc   10 ng/ml aTc   100 ng/ml aTc

Lane	Gene ID	Plasmid name	Gene name	Toxic/Non-toxic	Colony color on 100 ng/ml aTc plate*
+ Toxic control	--	pExTra02	Fruitloop *52*	Toxic	-
- Non-toxic control	--	pExTra03	Fruitloop *52* mutant	Non-toxic	+
1		pExTra-Lebron110	Lebron 110 replicate 1	Non-toxic	-
2		pExTra-Lebron110	Lebron 110 replicate 2	Non-toxic	-
3		pExTra-Lebron110	Lebron 110 replicate 3	Non-toxic	+

*Key: NG (no growth) - (no pink color) +(faint pink color) ++(obvious pink color) +++ (dark pink color)

Gene *111*; Score *0*

Images taken after 5 days at 37 ºC

- + 1 2 3   - + 1 2 3   - + 1 2 3

Dilution factor: 10^{0}, 10^{-1}, 10^{-2}, 10^{-3}, 10^{-4}, 10^{-5}

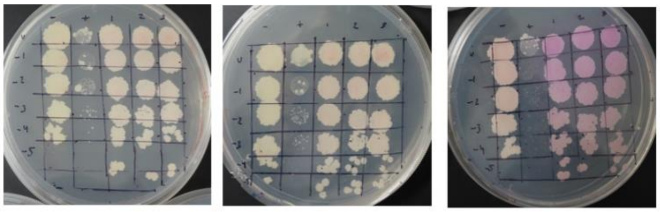

0 ng/ml aTc   10 ng/ml aTc   100 ng/ml aTc

Lane	Gene ID	Plasmid name	Gene name	Toxic/Non-toxic	Colony color on 100 ng/ml aTc plate*
- Non-toxic control	--	pExTra03	Fruitloop 52 mutant	Non-toxic	+
+ Toxic control	--	pExTra02	Fruitloop *52*	Toxic	-
1	–	pExTra-Lebron 111	Lebron *111* replicate 1	Non-toxic	+++
2	–	pExTra-Lebron 111	Lebron *111* replicate 2	Non-toxic	+++
3	–	pExTra-Lebron 111	Lebron *111* replicate 3	Non-toxic	+++

*Key: NG (no growth) - (no pink color) +(faint pink color) ++(obvious pink color) +++ (dark pink color)

Gene *113*; Score *1*

Images taken after 5 days at 37 ºC

- + 1 2 3   - + 1 2 3   - + 1 2 3

Dilution factor: 10^{0}, 10^{-1}, 10^{-2}, 10^{-3}, 10^{-4}, 10^{-5}

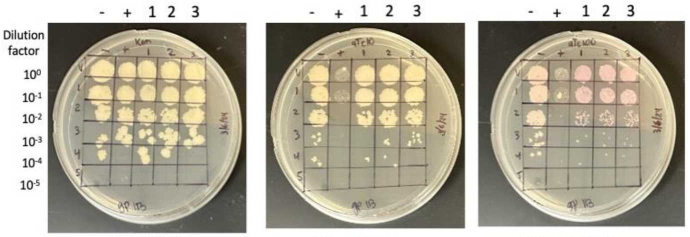

0 ng/ml aTc   10 ng/ml aTc   100 ng/ml aTc

Lane	Gene ID	Plasmid name	Gene name	Toxic/Non-toxic	Colony color on 100 ng/ml aTc plate*
- Non-toxic control	--	pExTra03	Fruitloop *52* mutant	Non-toxic	+
+ Toxic control	--	pExTra02	Fruitloop *52*	Toxic	-
1		pExTra-Lebron 113	Lebron *113* replicate 1	Toxic	++
2		pExTra-Lebron 113	Lebron *113* replicate 2	Toxic	++
3		pExTra-Lebron 113	Lebron *113* replicate 3	Toxic	++

*Key: NG (no growth) - (no pink color) +(faint pink color) ++(obvious pink color) +++ (dark pink color)

Gene 115; Score *0*

Images taken after 5 days at 37 ºC

\- + 1 2 3 | - + 1 2 3 | - + 1 2 3

Dilution factor: 10^0, 10^{-1}, 10^{-2}, 10^{-3}, 10^{-4}, 10^{-5}

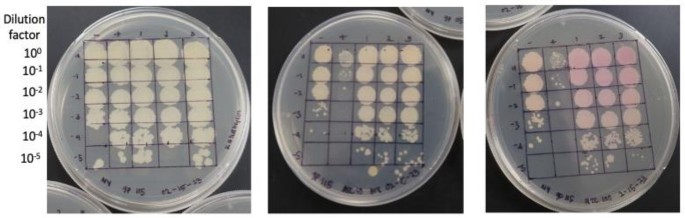

0 ng/ml aTc | 10 ng/ml aTc | 100 ng/ml aTc

Lane	Gene ID	Plasmid name	Gene name	Toxic/Non-toxic	Colony color on 100 ng/ml aTc plate*
+ Toxic control	--	pExTra02	Fruitloop *52*	Toxic	-
- Non-toxic control	--	pExTra03	Fruitloop *52* mutant	Non-toxic	+
1		pExTra-Lebron 115	Lebron 115 replicate 1	Non-toxic	++
2		pExTra-Lebron 115	Lebron 115 replicate 2	Non-toxic	++
3		pExTra-Lebron 115	Lebron 115 replicate 3	Non-toxic	++

*Key: NG (no growth) - (no pink color) +(faint pink color) ++(obvious pink color) +++ (dark pink color)

Gene *116*; Score *0*

Images taken after 5 days at 37 ºC

\- + 1 2 3 | - + 1 2 3 | - + 1 2 3

Dilution factor: 10^0, 10^{-1}, 10^{-2}, 10^{-3}, 10^{-4}, 10^{-5}

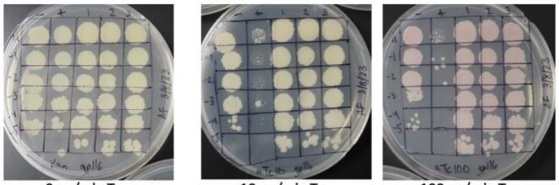

0 ng/ml aTc | 10 ng/ml aTc | 100 ng/ml aTc

Lane	Gene ID	Plasmid name	Gene name	Toxic/Non-toxic	Colony color on 100 ng/ml aTc plate*
+ Toxic control	--	pExTra02	Fruitloop *52*	Toxic	-
- Non-toxic control	--	pExTra03	Fruitloop *52* mutant	Non-toxic	+
1		pExTra-Lebron *116*	Lebron *116* replicate 1	Non-toxic	+
2		pExTra-Lebron *116*	Lebron *116* replicate 2	Non-toxic	+
3		pExTra-Lebron *116*	Lebron *116* replicate 3	Non-toxic	+

*Key: NG (no growth) - (no pink color) +(faint pink color) ++(obvious pink color) +++ (dark pink color)

Gene *117*; Score *1*

Images taken after 5 days at 37 ºC

\- + 1 2 3 | - + 1 2 3 | - + 1 2 3

Dilution factor: 10^0, 10^{-1}, 10^{-2}, 10^{-3}, 10^{-4}, 10^{-5}

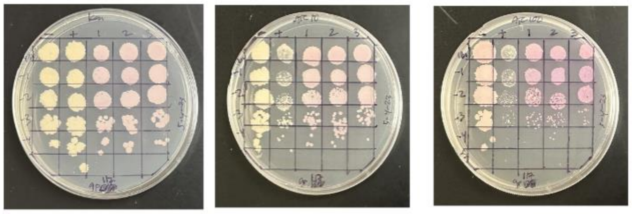

0 ng/ml aTc | 10 ng/ml aTc | 100 ng/ml aTc

Lane	Gene ID	Plasmid name	Gene name	Toxic/Non-toxic	Colony color on 100 ng/ml aTc plate*
- Non-toxic control	--	pExTra03	Fruitloop *52* mutant	Non-toxic	+
+ Toxic control	--	pExTra02	Fruitloop *52*	Toxic	-
1		pExTra-Lebron 117	Lebron *117* replicate 1	Toxic	++
2		pExTra-Lebron 117	Lebron *117* replicate 2	Toxic	++
3		pExTra-Lebron 117	Lebron *117* replicate 3	Toxic	++

*Key: NG (no growth) - (no pink color) +(faint pink color) ++(obvious pink color) +++ (dark pink color)

Gene *118*; Score *0*

Images taken after 5 days at 37 ºC

\- + 1 2 3 | - + 1 2 3 | - + 1 2 3

Dilution factor: 10^0, 10^{-1}, 10^{-2}, 10^{-3}, 10^{-4}, 10^{-5}

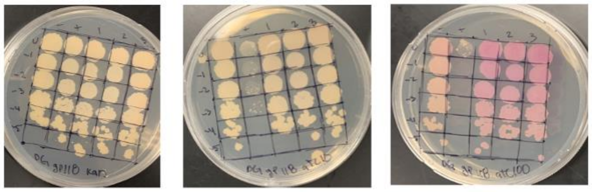

0 ng/ml aTc | 10 ng/ml aTc | 100 ng/ml aTc

Lane	Gene ID	Plasmid name	Gene name	Toxic/Non-toxic	Colony color on 100 ng/ml aTc plate*
+ Toxic control	--	pExTra02	Fruitloop *52*	Toxic	-
- Non-toxic control	--	pExTra03	Fruitloop *52* mutant	Non-toxic	++
1	131436	pExTra-Lebron 118	Lebron 118 replicate 1	Non-toxic	++
2	131436	pExTra-Lebron 118	Lebron 118 replicate 2	Non-toxic	++
3	131436	pExTra-Lebron 118	Lebron 118 replicate 3	Non-toxic	+++

*Key: NG (no growth) - (no pink color) +(faint pink color) ++(obvious pink color) +++ (dark pink color)

Gene 119; Score *1*

Images taken after 5 days at 37 ºC

\- + 1 2 3 | - + 1 2 3 | - + 1 2 3

Dilution factor: 10^0, 10^{-1}, 10^{-2}, 10^{-3}, 10^{-4}, 10^{-5}

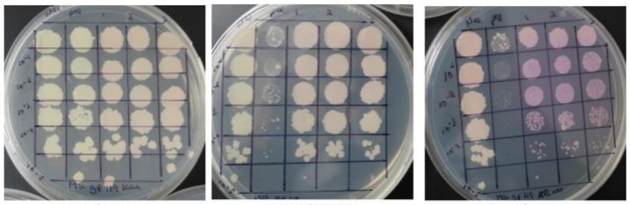

0 ng/ml aTc | 10 ng/ml aTc | 100 ng/ml aTc

Lane	Gene ID	Plasmid name	Gene name	Toxic/Non-toxic	Colony color on 100 ng/ml aTc plate*
+ Toxic control	--	pExTra02	Fruitloop *52*	Toxic	-
- Non-toxic control	--	pExTra03	Fruitloop *52* mutant	Non-toxic	+
1		pExTra-Lebron *119*	Lebron *119* replicate 1	toxic	++
2		pExTra-Lebron *119*	Lebron *119* replicate 2	toxic	++
3		pExTra-Lebron *119*	Lebron *119* replicate 3	toxic	++

*Key: NG (no growth) - (no pink color) +(faint pink color) ++(obvious pink color) +++ (dark pink color)

Gene *120*; Score *0*

Images taken after 5 days at 37 ºC

\- + 1 2 3 | - + 1 2 3 | - + 1 2 3

Dilution factor: 10^0, 10^{-1}, 10^{-2}, 10^{-3}, 10^{-4}, 10^{-5}

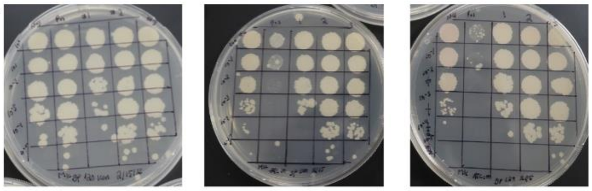

0 ng/ml aTc | 10 ng/ml aTc | 100 ng/ml aTc

Lane	Gene ID	Plasmid name	Gene name	Toxic/Non-toxic	Colony color on 100 ng/ml aTc plate*
+ Toxic control	--	pExTra02	Fruitloop 52	Toxic	-
- Non-toxic control	--	pExTra03	Fruitloop 52 mutant	Non-toxic	+
1		pExTra-Lebron 120	Lebron *120* replicate 1	Non-toxic	-
2		pExTra-Lebron 120	Lebron *120* replicate 2	Non-toxic	-
3		pExTra-Lebron 120	Lebron *120* replicate 3	Non-toxic	-

*Key: NG (no growth) - (no pink color) +(faint pink color) ++(obvious pink color) +++ (dark pink color)

Gene *121*; Score *0*

Images taken after 5 days at 37 ºC

\- + 1 2 3 | - + 1 2 3 | - + 1 2 3

Dilution factor: 10^0, 10^{-1}, 10^{-2}, 10^{-3}, 10^{-4}, 10^{-5}

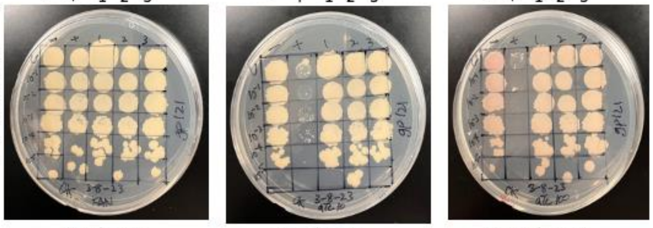

0 ng/ml aTc | 10 ng/ml aTc | 100 ng/ml aTc

Lane	Gene ID	Plasmid name	Gene name	Toxic/Non-toxic	Colony color on 100 ng/ml aTc plate*
- Non-toxic control	--	pExTra03	Fruitloop *52* mutant	Non-toxic	+
+ Toxic control	--	pExTra02	Fruitloop *52*	Toxic	–
1	–	pExTra-Lebron 121	Lebron *121* replicate 1	Non-toxic	+
2	–	pExTra-Lebron 121	Lebron *121* replicate 2	Non-toxic	+
3	–	pExTra-Lebron 121	Lebron *121* replicate 3	Non-toxic	+

*Key: NG (no growth) - (no pink color) +(faint pink color) ++(obvious pink color) +++ (dark pink color)

Gene *122*; Score *0*

Images taken after 5 days at 37 ºC

\- + 1 2 3 | - + 1 2 3 | - + 1 2 3

Dilution factor: 10^0, 10^{-1}, 10^{-2}, 10^{-3}, 10^{-4}, 10^{-5}

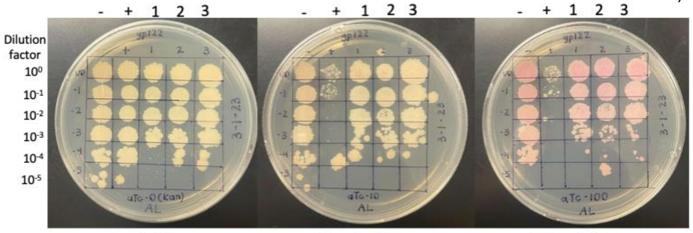

0 ng/ml aTc | 10 ng/ml aTc | 100 ng/ml aTc

Lane	Gene ID	Plasmid name	Gene name	Toxic/Non-toxic	Colony color on 100 ng/ml aTc plate*
+ Toxic control	--	pExTra02	Fruitloop *52*	Toxic	-
- Non-toxic control	--	pExTra03	Fruitloop *52* mutant	Non-toxic	++
1		pExTra-Lebron122	Lebron 122 replicate 1	Non-toxic	++
2		pExTra-Lebron122	Lebron 122 replicate 2	Non-toxic	++
3		pExTra-Lebron122	Lebron 122 replicate 3	Non-toxic	++

*Key: NG (no growth) - (no pink color) +(faint pink color) ++(obvious pink color) +++ (dark pink color)

Gene 123: Score *0*

Images taken after 5 days at 37 ºC

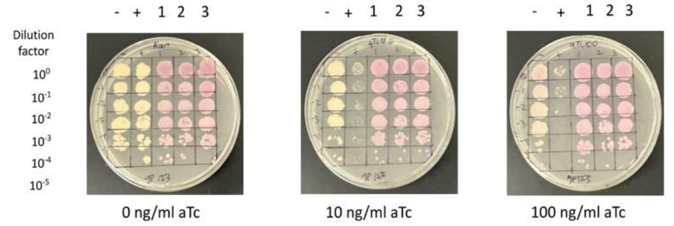

Lane	Gene ID	Plasmid name	Gene name	Toxic/Non-toxic	Colony color on 100 ng/ml aTc plate*
- Non-toxic control	--	pExTra03	Fruitloop *52* mutant	Non-toxic	+
+ Toxic control	--	pExTra02	Fruitloop *52*	Toxic	-
1		pExTra-Lebron 123	Lebron *123* replicate 1	Non-toxic	++
2		pExTra-Lebron 123	Lebron *123* replicate 2	Non-toxic	++
3		pExTra-Lebron 123	Lebron *123* replicate 3	Non-toxic	++

*Key: NG (no growth) - (no pink color) +(faint pink color) ++(obvious pink color) +++ (dark pink color)

Gene *124*; Score *0*

Images taken after 5 days at 37 ºC

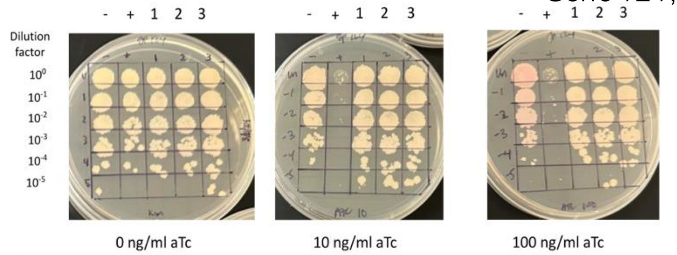

Lane	Gene ID	Plasmid name	Gene name	Toxic/Non-toxic	Colony color on 100 ng/ml aTc plate*
- Non-toxic control	--	pExTra03	Fruitloop *52* mutant	Non-toxic	++
+ Toxic control	--	pExTra02	Fruitloop *52*	Toxic	-
1		pExTra-Lebron 124	Lebron *124* replicate 1	Non-toxic	+
2		pExTra-Lebron 124	Lebron *124* replicate 2	Non-toxic	+
3		pExTra-Lebron 124	Lebron *124* replicate 3	Non-toxic	+

*Key: NG (no growth) - (no pink color) +(faint pink color) ++(obvious pink color) +++ (dark pink color)

Gene *125*; Score *1*

Images taken after 5 days at 37 ºC

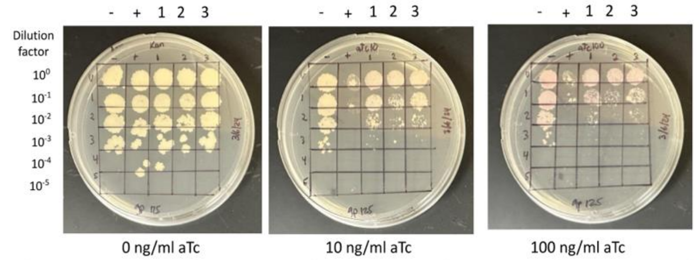

Lane	Gene ID	Plasmid name	Gene name	Toxic/Non-toxic	Colony color on 100 ng/ml aTc plate*
- Non-toxic control	--	pExTra03	Fruitloop *52* mutant	Non-toxic	+
+ Toxic control	--	pExTra02	Fruitloop *52*	Toxic	-
1		pExTra-Lebron 125	Lebron *125* replicate 1	Toxic	+
2		pExTra-Lebron 125	Lebron *125* replicate 2	Toxic	+
3		pExTra-Lebron 125	Lebron *125* replicate 3	Toxic	+

*Key: NG (no growth) - (no pink color) +(faint pink color) ++(obvious pink color) +++ (dark pink color)

Gene *126*; Score *0*

Images taken after 5 days at 37 ºC

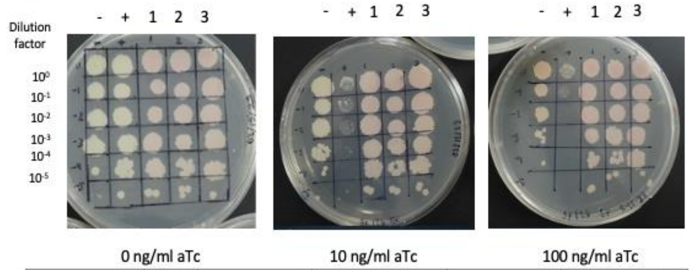

Lane	Gene ID	Plasmid name	Gene name	Toxic/Non-toxic	Colony color on 100 ng/ml aTc plate*
- Non-toxic control	--	pExTra03	Fruitloop *52* mutant	Non-toxic	+
+ Toxic control	--	pExTra02	Fruitloop *52*	Toxic	-
1	--	pExTra-Lebron 126	Lebron *126* replicate 1	Non-toxic	+
2	--	pExTra-Lebron 126	Lebron *126* replicate 2	Non-toxic	+
3	--	pExTra-Lebron 126	Lebron *126* replicate 3	Non-toxic	+

*Key: NG (no growth) - (no pink color) +(faint pink color) ++(obvious pink color) +++ (dark pink color)

Gene 127; Score *0*

Images taken after 5 days at 37 ºC

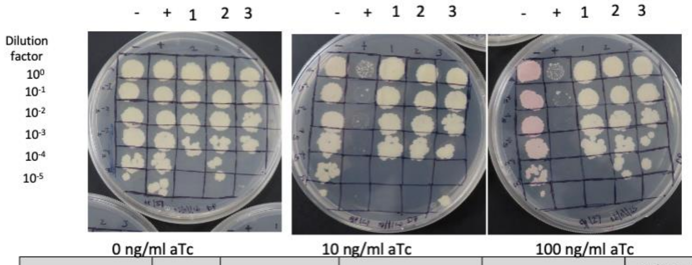

Lane	Gene ID	Plasmid name	Gene name	Toxic/Non-toxic	Colony color on 100 ng/ml aTc plate*
+ Toxic control	--	pExTra02	Fruitloop *52*	Toxic	-
- Non-toxic control	--	pExTra03	Fruitloop *52* mutant	Non-toxic	++
1	--	pExTra-Lebron 127	Lebron *127* replicate 1	Non-toxic	-
2	--	pExTra-Lebron 127	Lebron *127* replicate 2	Non-toxic	-
3	--	pExTra-Lebron 127	Lebron *127* replicate 3	Non-toxic	-

*Key: NG (no growth) - (no pink color) +(faint pink color) ++(obvious pink color) +++ (dark pink color)

Gene *128*; Score *3*

Images taken after 5 days at 37 ºC

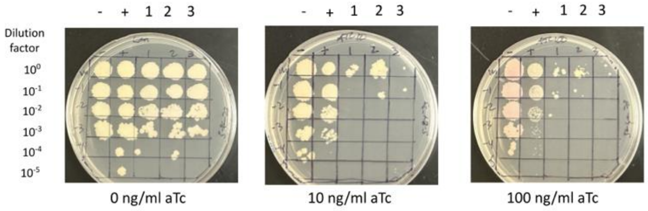

Lane	Gene ID	Plasmid name	Gene name	Toxic/Non-toxic	Colony color on 100 ng/ml aTc plate*
- Non-toxic control	--	pExTra03	Fruitloop *52* mutant	Non-toxic	+
+ Toxic control	--	pExTra02	Fruitloop *52*	Toxic	-
1		pExTra-Lebron 128	Lebron *128* replicate 1	Toxic	-
2		pExTra-Lebron 128	Lebron *128* replicate 2	Toxic	-
3		pExTra-Lebron 128	Lebron *128* replicate 3	Toxic	-

*Key: NG (no growth) - (no pink color) +(faint pink color) ++(obvious pink color) +++ (dark pink color)

Gene *129*; Score *0*

Images taken after 5 days at 37 ºC

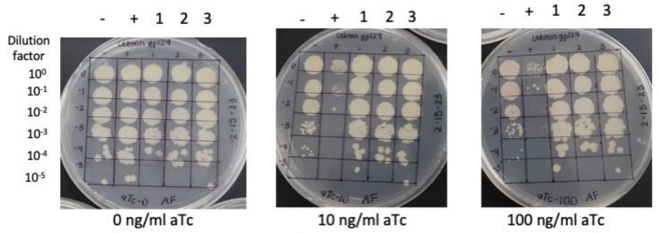

Lane	Gene ID	Plasmid name	Gene name	Toxic/Non-toxic	Colony color on 100 ng/ml aTc plate*
+ Toxic control	--	pExTra02	Fruitloop *52*	Toxic	-
- Non-toxic control	--	pExTra03	Fruitloop *52* mutant	Non-toxic	+
1	131436	pExTra-Lebron *129*	Lebron *129* replicate 1	Non-toxic	-
2	131436	pExTra-Lebron *129*	Lebron *129* replicate 2	Non-toxic	-
3	131436	pExTra-Lebron *129*	Lebron *129* replicate 3	Non-toxic	-

*Key: NG (no growth) - (no pink color) +(faint pink color) ++(obvious pink color) +++ (dark pink color)

Gene *130*; Score *0*

Images taken after 5 days at 37 ºC

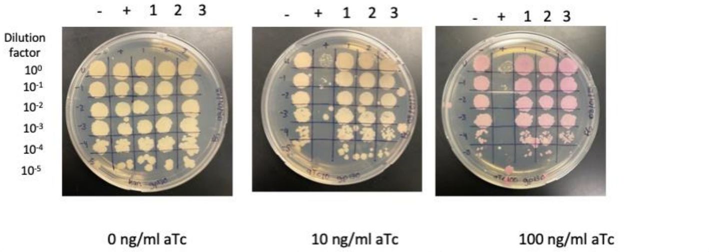

Lane	Gene ID	Plasmid name	Gene name	Toxic/Non-toxic	Colony color on 100 ng/ml aTc plate*
+ Toxic control	--	pExTra02	Fruitloop *52*	Toxic	-
- Non-toxic control	--	pExTra03	Fruitloop *52* mutant	Non-toxic	++
1		pExTra-Lebron 130	Lebron 130 replicate 1	Non-Toxic	+++
2		pExTra-Lebron 130	Lebron 130 replicate 2	Non-Toxic	+++
3		pExTra-Lebron 130	Lebron *130* replicate 3	Non-Toxic	+++

*Key: NG (no growth) - (no pink color) +(faint pink color) ++(obvious pink color) +++ (dark pink color)

Gene 131: Score *1*

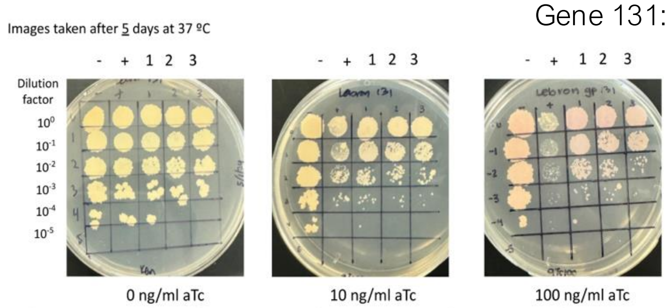

Lane	Gene ID	Plasmid name	Gene name	Toxic/Non-toxic	Colony color on 100 ng/ml aTc plate*
- Non-toxic control	--	pExTra03	Fruitloop *52* mutant	Non-toxic	+
+ Toxic control	--	pExTra02	Fruitloop *52*	Toxic	-
1		pExTra-Lebron 131	Lebron *131* replicate 1	Toxic	++
2		pExTra-Lebron 131	Lebron *131* replicate 2	Toxic	++
3		pExTra-Lebron 131	Lebron *131* replicate 3	Toxic	++

*Key: NG (no growth) - (no pink color) +(faint pink color) ++(obvious pink color) +++ (dark pink color)

Gene 132: Score *0*

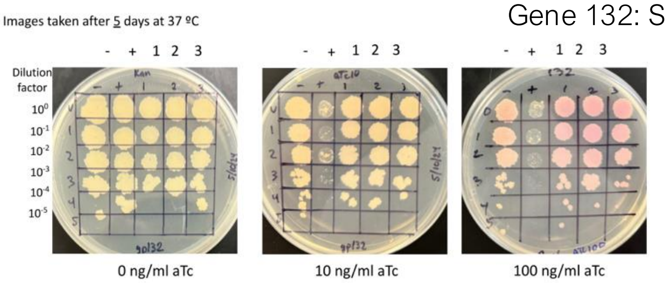

Lane	Gene ID	Plasmid name	Gene name	Toxic/Non-toxic	Colony color on 100 ng/ml aTc plate*
- Non-toxic control	--	pExTra03	Fruitloop *52* mutant	Non-toxic	+
+ Toxic control	--	pExTra02	Fruitloop *52*	Toxic	-
1		pExTra-Lebron 132	Lebron *132* replicate 1	Non-toxic	+++
2		pExTra-Lebron 132	Lebron *132* replicate 2	Non-toxic	+++
3		pExTra-Lebron 132	Lebron *132* replicate 3	Non-toxic	+++

*Key: NG (no growth) - (no pink color) +(faint pink color) ++(obvious pink color) +++ (dark pink color)

Supplementary Figure 1. Shown are the results of representative cytotoxicity assays for the 122 LeBron genes screened in this study. Each strain was spotted in triplicate alongside *M. smegmatis*/pExtra-Fruitloop52 (+) and pExtra-Fruitloop52I170S (-) control strain in 7H10 Kan supplemented with 0, 10, 100 ng/ml aTc. In all experiments, 10^0 to 10^{-5} dilutions are shown. Plates were monitored over 5 days at 37ºC, with results shown to best illustrate effects on colony color and size. Colony color was scored using the indicated key shown at the bottom of the data card.
