# Supplementary material for: A systematic overexpression screen identifies cytotoxic genes encoded by the Cluster L1 mycobacteriophage LeBron
Source: G3 (Bethesda). 2026 Feb 19;16(5):jkag045. doi: 10.1093/g3journal/jkag045 (PMC13148394; doi:10.1093/g3journal/jkag045)
Supplement: jkag045_Supplementary_Data [file jkag045_supplementary_data.zip › Supplementary_Table_1_G3-2025-406480.pdf]

Supplementary Table 1: Primer sequences used in this study to amplify genes

Primer Name	Primer Sequence (5' to 3')
oLebron1_F	atgcggaggaatcacttccatATGGGTAGAGGACGTGGTAAGG
oLebron1_R	tgcaggatccgactcgagtgtcgacTCACTTGCCGTACTTCTCAAG
oLebron2_F	atgcggaggaatcacttccatATGGGGGATGGCGTTAAAAAC
oLebron2_R	tgcaggatccgactcgagtgtcgacTCAGGACGCCTGAGCG
oLebron3_F	atgcggaggaatcacttccatATGCAGTCCATTCGTTCGG
oLebron3_R	tgcaggatccgactcgagtgtcgacTCAATCATCACCATTACCAATCTC
oLebron4_F	atgcggaggaatcacttccatATGACGGTTATTCCGTCAATTCCC
oLebron4_R	tgcaggatccgactcgagtgtcgacTCAATAGCCATTAGTCGACCCC
oLebron5_F	atgcggaggaatcacttccatATGGCTATTGAGCTACCCG
oLebron5_R	tgcaggatccgactcgagtgtcgacTCAGGCAGCCTTACTGGC
oLebron6_F	atgcggaggaatcacttccatATGGCTGAGGCTGACGAG
oLebron6_R	tgcaggatccgactcgagtgtcgacTCAAGCTGAAGCGCTTAGTTTCC
oLebron7_F	atgcggaggaatcacttccatATGACTGGCTTAAGTGAAGGAAAAT
oLebron7_R	tgcaggatccgactcgagtgtcgacTCAGACGCCGCCGCG
oLebron8_F	atgcggaggaatcacttccatATGGCGCACATTTTTGTGAAGC
oLebron8_R	tgcaggatccgactcgagtgtcgacTCAGGCAGGGACGGTG
oLebron9_F	atgcggaggaatcacttccatATGCAGACCCTCATGTCG
oLebron9_R	tgcaggatccgactcgagtgtcgacTCACCAGGGCACCACC
oLebron10_F	atgcggaggaatcacttccatATGAACGAAGAGACCATAACGG
oLebron10_R	tgcaggatccgactcgagtgtcgacTCACCCCGTCACCGCC
oLebron11_F	atgcggaggaatcacttccatATGCCTGCTGCGGGTAC
oLebron11_R	tgcaggatccgactcgagtgtcgacTCACAGCCCATTCTTAGCC
oLebron12_F	atgcggaggaatcacttccatATGGCTCTGGCGCTGC
oLebron12_R	tgcaggatccgactcgagtgtcgacTCAAGAACGAAACAGCTCATAAATG
oLebron13_F	atgcggaggaatcacttccatATGGCTGATTTCTACACGATTAAGG
oLebron13_R	tgcaggatccgactcgagtgtcgacTCACGCCGAGACGGTC
oLebron14_F	atgcggaggaatcacttccatATGGCTGAAAAGAGCAAGG
oLebron14_R	tgcaggatccgactcgagtgtcgacTCACTTTCCCGAGTCCTTATCC
oLebron15_F	atgcggaggaatcacttccatATGGCTGAAAAGAGCAAGG
oLebron15_R	tgcaggatccgactcgagtgtcgacTCATAGGGTGACCCATTTCG
oLebron16_F	atgcggaggaatcacttccatATGGCTGAATACGTTGCC
oLebron16_R	tgcaggatccgactcgagtgtcgacTCATCGCATTGGCAGGATGG
oLebron17_F	atgcggaggaatcacttccatATGGAAAGACAACTGAATATGTCCG
oLebron17_R	tgcaggatccgactcgagtgtcgacTCAGCGCAGCACCCCC
oLebron18_F	atgcggaggaatcacttccatATGGCTGACACGCAGTATTGG
oLebron18_R	tgcaggatccgactcgagtgtcgacTCAACTCTGGATAATGTGGACACC
oLebron19_F	atgcggaggaatcacttccatATGGCTAAAACGCAGTCGG

oLebron19_R	tgcaggatccgactcgagtgtcgacTCAGGTCAACCTCCCCTCC
oLebron20_F	atgcggaggaatcacttccatATGACGATGCCTAACGGGG
oLebron20_R	tgcaggatccgactcgagtgtcgacTCAATTACCAGACAAATCCATCGCC
oLebron21_F	atgcggaggaatcacttccatATGATGACGTGGGCGG
oLebron21_R	tgcaggatccgactcgagtgtcgacTCAAGAATTGCCCCATTGAAAGG
oLebron22_F	atgcggaggaatcacttccatATGTTGGATCACGCGTTCAGG
oLebron22_R	tgcaggatccgactcgagtgtcgacTCAAGCCGCAATCGGC
oLebron23_F	atgcggaggaatcacttccatATGAACTATCAGGCTGGCC
oLebron23_R	tgcaggatccgactcgagtgtcgacTCAAGCATTGAACCACCCC
oLebron24_F	atgcggaggaatcacttccatATGCTTAACATCGGCCCG
oLebron24_R	tgcaggatccgactcgagtgtcgacTCAAACACCGTCCTTCGC
oLebron25_F	atgcggaggaatcacttccatATGGCGCTACTGGGAAGC
oLebron25_R	tgcaggatccgactcgagtgtcgacTCAGCTCGCCGCTTTCAG
oLebron26_F	atgcggaggaatcacttccatATGGCGAAGGGTCTCAATG
oLebron26_R	tgcaggatccgactcgagtgtcgacTCAAACAACACTCCTTAGGTAATC
oLebron27_F	atgcggaggaatcacttccatATGTTGGTTTACGACGAGAAG
oLebron27_R	tgcaggatccgactcgagtgtcgacTCACTCACCGTCCTTACC
oLebron28_F	atgcggaggaatcacttccatATGAAGATTCTCGGTCACAAGC
oLebron28_R	tgcaggatccgactcgagtgtcgacTCATCCCTTCTCAATAAAGGGCG
oLebron29_F	atgcggaggaatcacttccatATGGACCCGAGCGATATGG
oLebron29_R	tgcaggatccgactcgagtgtcgacTCACTTCCAAATGTCCAATTCC
oLebron30_F	atgcggaggaatcacttccatATGGAAGTAAAAGTTTATTCGCCG
oLebron30_R	tgcaggatccgactcgagtgtcgacTCAGGCCGCTGTCTGG
oLebron31_F	atgcggaggaatcacttccatATGATTGAGGCTGTGGTGC
oLebron31_R	tgcaggatccgactcgagtgtcgacTCAGAGTTTGGCGGGTGG
oLebron32_F	atgcggaggaatcacttccatATGCAGCCGCTGATCATTGG
oLebron32_R	tgcaggatccgactcgagtgtcgacTCATGCCGCCAGTTTATCTC
oLebron33_F	atgcggaggaatcacttccatATGGGTAAGCGCGTAACC
oLebron33_R	tgcaggatccgactcgagtgtcgacTCAGGACTGCGCTGCG
oLebron34_F	atgcggaggaatcacttccatATGAGAATCCGTTGGCC
oLebron34_R	tgcaggatccgactcgagtgtcgacTCACCCATCGCTTTCGTATCC
oLebron35_F	atgcggaggaatcacttccatATGCCTGGTCAGAGCGG
oLebron35_R	tgcaggatccgactcgagtgtcgacTCACTCAACCCCCTGC
oLebron36_F	atgcggaggaatcacttccatATGGCCGAGACGGACTC
oLebron36_R	tgcaggatccgactcgagtgtcgacTCAAACACTGGGTAACAGAGC
oLebron37_F	atgcggaggaatcacttccatATGCATACGACGCACTCTG
oLebron37_R	tgcaggatccgactcgagtgtcgacTCAGTGCGCGGTCTTGTG
oLebron38_F	atgcggaggaatcacttccatATGCGCATGTCCCAAAAATCG
oLebron38_R	tgcaggatccgactcgagtgtcgacTCACAGGGGAGGAATATCTG

oLebron39_F	atgcggaggaatcacttccatATGCCTTCCGAACTTTGTCG
oLebron39_R	tgcaggatccgactcgagtgtcgacTCACACGGCCCTCCGG
oLebron40_F	atgcggaggaatcacttccatATGACCGCGCCCACAG
oLebron40_R	tgcaggatccgactcgagtgtcgacTCACAGGTCTGCCTCC
oLebron41_F	atgcggaggaatcacttccatATGACCGCCGCGCAAG
oLebron41_R	tgcaggatccgactcgagtgtcgacTCACCCCTGATTGCCG
oLebron42_F	atgcggaggaatcacttccatATGTGCGACCCGGCAATC
oLebron42_R	tgcaggatccgactcgagtgtcgacTCATACCGTCCAGCCC
oLebron43_F	atgcggaggaatcacttccatATGAGTACAGCGGGTGTAAAAAATC
oLebron43_R	tgcaggatccgactcgagtgtcgacTCAGATGGGAGTCGCG
oLebron44_F	atgcggaggaatcacttccatATGCGCGGTTATCGGG
oLebron44_R	tgcaggatccgactcgagtgtcgacTCAGCTCTCCCTTCCC
oLebron45_F	atgcggaggaatcacttccatATGACCGTTATCTATTTGGGC
oLebron45_R	tgcaggatccgactcgagtgtcgacTCAGAGGTAGTCGTCGC
oLebron46_F	atgcggaggaatcacttccatATGGACAAAGACTGGCTGTTTTCAG
oLebron46_R	tgcaggatccgactcgagtgtcgacTCAGCCTTCATCGGGC
oLebron47_F	atgcggaggaatcacttccatATGAAGGCTAGCCAGCGG
oLebron47_R	tgcaggatccgactcgagtgtcgacTCAGCCCGCTTGAGGC
oLebron48_F	atgcggaggaatcacttccatATGCGAAACGCCGTATTGG
oLebron48_R	tgcaggatccgactcgagtgtcgacTCAGGGGAGGCTAGGAG
oLebron49_F	atgcggaggaatcacttccatATGGAAGGAACCATTTTCTTGAACC
oLebron49_R	tgcaggatccgactcgagtgtcgacTCAGCGAGCCGGGGCAC
oLebron50_F	atgcggaggaatcacttccatATGTCGCAAAAGAGCGTGTTCG
oLebron50_R	tgcaggatccgactcgagtgtcgacTCATCTCTTAGCCCCCACTCC
oLebron51_F	atgcggaggaatcacttccatATGATCGGTTCTGTTTGGCG
oLebron51_R	tgcaggatccgactcgagtgtcgacTCAAAAGCTGGCACCGTAAACC
oLebron52_F	atgcggaggaatcacttccatATGCCAGCTTTTGAGGGTG
oLebron52_R	tgcaggatccgactcgagtgtcgacTCATTTCCCGACGTAAAGG
oLebron53_F	atgcggaggaatcacttccatATGACGACATTCCTTGACAGTC
oLebron53_R	tgcaggatccgactcgagtgtcgacTCACAACGTGATCTGCGAGTACC
oLebron54_F	atgcggaggaatcacttccatATGGCTAAGACGGTTCGAGAG
oLebron54_R	tgcaggatccgactcgagtgtcgacTCACCGGCGCTCCACG
oLebron55_F	atgcggaggaatcacttccatATGGTGGTCCTGCTCC
oLebron55_R	tgcaggatccgactcgagtgtcgacTCAAAGCTCCCATCGAAAGC
oLebron56_F	atgcggaggaatcacttccatATGAGCGGCTACTACGAGG
oLebron56_R	tgcaggatccgactcgagtgtcgacTCATTCATCCCTGTATTCCTTAACG
oLebron57_F	atgcggaggaatcacttccatATGAATAAGCCGGTCGGC
oLebron57_R	tgcaggatccgactcgagtgtcgacTCATCCTTCAATGATCACAGTG
oLebron58_F	atgcggaggaatcacttccatATGGTTTACCCACCGGACTTTACC

oLebron58_R	tgcaggatccgactcgagtgtcgacTCACCAGTGATAACGGCC
oLebron59_F	atgcggaggaatcacttccatATGGCCGTTATCACTGGTAATCG
oLebron59_R	tgcaggatccgactcgagtgtcgacTCACTTAGCTCCGTTCTTAGCTTTC
oLebron60_F	atgcggaggaatcacttccatATGACAGCCACGAAAGCTG
oLebron60_R	tgcaggatccgactcgagtgtcgacTCAGAGAGTCACCATCCCC
oLebron61_F	atgcggaggaatcacttccatATGCCGCCTCCTGTTAAGG
oLebron61_R	tgcaggatccgactcgagtgtcgacTCATGTGAGACCTTGCCG
oLebron62_F	atgcggaggaatcacttccatATGCGTGGCATGTCTATTCC
oLebron62_R	tgcaggatccgactcgagtgtcgacTCAACCGGCTAGCGCAAAG
oLebron63_F	atgcggaggaatcacttccatATGAGCGATACCTACGAGTACAACC
oLebron63_R	tgcaggatccgactcgagtgtcgacTCACCATTCACCCCTGC
oLebron64_F	atgcggaggaatcacttccatATGGTGAAAAAGATATTTGCGGC
oLebron64_R	tgcaggatccgactcgagtgtcgacTCATGGCTCTTCCGGTTCATC
oLebron65_F	atgcggaggaatcacttccatATGTGGACCCCCGATAAC
oLebron65_R	tgcaggatccgactcgagtgtcgacTCACTGCACTGGCCCAC
oLebron66_F	atgcggaggaatcacttccatATGAGCGACTTCGGGAAAATCC
oLebron66_R	tgcaggatccgactcgagtgtcgacTCAAGCCGCTACCCTTTCC
oLebron67_F	atgcggaggaatcacttccatATGACGGAGGCTCAGC
oLebron67_R	tgcaggatccgactcgagtgtcgacTCAAGTGATTCGCTGGTAGTGC
oLebron68_F	atgcggaggaatcacttccatATGGAGGGCAAGCCGG
oLebron68_R	tgcaggatccgactcgagtgtcgacTCATGCTGAATTGTCACGCG
oLebron69_F	atgcggaggaatcacttccatATGGAGATGGCTAAAGCCAAGG
oLebron69_R	tgcaggatccgactcgagtgtcgacTCACCATTTGTCCCCCAACC
oLebron70_F	atgcggaggaatcacttccatATGGTAGATCCCGATGAGGAC
oLebron70_R	tgcaggatccgactcgagtgtcgacTCAGATAAACGGCCATGAATTCGG
oLebron71_F	atgcggaggaatcacttccatATGGCCGTTTATCTGAGGGC
oLebron71_R	tgcaggatccgactcgagtgtcgacTCAGTGCTCACTTAACCCCTTAACC
oLebron72_F	atgcggaggaatcacttccatATGAGCACTAAGTTCGCGCC
oLebron72_R	tgcaggatccgactcgagtgtcgacTCATTGGCTACTCGCCAAAAC
oLebron73_F	atgcggaggaatcacttccatATGGCAACCACGCCGAAG
oLebron73_R	tgcaggatccgactcgagtgtcgacTCACTGGATACCGGCTTTACC
oLebron74_F	atgcggaggaatcacttccatATGAGCGAGCTGTCGTTAAC
oLebron74_R	tgcaggatccgactcgagtgtcgacTCAGAAATCTCCCGGCTGAAC
oLebron75_F	atgcggaggaatcacttccatATGAAACTGGGAAACGTCGTG
oLebron75_R	tgcaggatccgactcgagtgtcgacTCACTTGAACTCGATAGACACCTGC
oLebron76_F	atgcggaggaatcacttccatATGGAGCACCTACTTGTAACG
oLebron76_R	tgcaggatccgactcgagtgtcgacTCAGCCAGCTTGCGCC
oLebron77_F	atgcggaggaatcacttccatATGTTTTTCCCCCTAAAGGGC
oLebron77_R	tgcaggatccgactcgagtgtcgacTCACGCAACCTCCATCAAATCG

oLebron78_F	atgcggaggaatcacttccatATGGCAGAACACGTGACGG
oLebron78_R	tgcaggatccgactcgagtgtcgacTCAAGCAGCATGTCGCTC
oLebron79_F	atgcggaggaatcacttccatATGCTGCTTAGCACCAAAATTAAGC
oLebron79_R	tgcaggatccgactcgagtgtcgacTCACGCATACCGTGGCAAC
oLebron80_F	atgcggaggaatcacttccatATGGGACTAGGGGAGC
oLebron80_R	tgcaggatccgactcgagtgtcgacTCAGCCACGCTTAACCTTCTC
oLebron81_F	atgcggaggaatcacttccatATGGCTAGTGTTGACATTCAG
oLebron81_R	tgcaggatccgactcgagtgtcgacTCAGAACGGAGGCTCTTCC
oLebron82_F	atgcggaggaatcacttccatATGCTGCGCAAAATCGG
oLebron82_R	tgcaggatccgactcgagtgtcgacTCATTCCCGCCACCAG
oLebron83_F	atgcggaggaatcacttccatATGATCGGGTTCCTGATTTTCC
oLebron83_R	tgcaggatccgactcgagtgtcgacTCATTCTTCGTCGTCCTCCTTAGC
oLebron84_F	atgcggaggaatcacttccatATGAAGAGAATCCTAGTAACCGG
oLebron84_R	tgcaggatccgactcgagtgtcgacTCAGTTGTCTCCGTAGTTGATTACG
oLebron85_F	atgcggaggaatcacttccatATGAGTTTTGACGCGCC
oLebron85_R	tgcaggatccgactcgagtgtcgacTCAAGCCGCTACCTCCATATCC
oLebron86_F	atgcggaggaatcacttccatATGAGTTACGACTACAAGGCTCC
oLebron86_R	tgcaggatccgactcgagtgtcgacTCAGCCGAGCACCACC
oLebron87_F	atgcggaggaatcacttccatATGGTCAATTACCTGTCGGG
oLebron87_R	tgcaggatccgactcgagtgtcgacTCATTCCTCTGCCCTCC
oLebron88_F	atgcggaggaatcacttccatATGAGTTACACGCTGTTCGC
oLebron88_R	tgcaggatccgactcgagtgtcgacTCAGGCGCGGACATGG
oLebron89_F	atgcggaggaatcacttccatATGTCCGCGCCTAAGTGG
oLebron89_R	tgcaggatccgactcgagtgtcgacTCACAAGTTCCCTTTGGTTGC
oLebron90_F	atgcggaggaatcacttccatATGTGGGTTGAGAGTCC
oLebron90_R	tgcaggatccgactcgagtgtcgacTCAAAGCACTTCCGGAAGC
oLebron91_F	atgcggaggaatcacttccatATGAGGCCGTCAGATGTTGC
oLebron91_R	tgcaggatccgactcgagtgtcgacTCAATTCTTTCCCCCTAAGC
oLebron92_F	atgcggaggaatcacttccatATGACCACGAGGGATGC
oLebron92_R	tgcaggatccgactcgagtgtcgacTCACGCGAACGCCCTC
oLebron93_F	atgcggaggaatcacttccatATGAGCGCGCGGGTCC
oLebron93_R	tgcaggatccgactcgagtgtcgacTCACACCGGCCTCAAC
oLebron94_F	atgcggaggaatcacttccatATGAGTTACATAAGCTTGGATCG
oLebron94_R	tgcaggatccgactcgagtgtcgacTCACCAAGCCTTCCCG
oLebron95_F	atgcggaggaatcacttccatATGATGGACGATCCTTACC
oLebron95_R	tgcaggatccgactcgagtgtcgacTCACTCATCGAGGTCAACC
oLebron96_F	atgcggaggaatcacttccatATGGAGACGACGGAGG
oLebron96_R	tgcaggatccgactcgagtgtcgacTCAAGCCGCTTGCGCAG
oLebron97_F	atgcggaggaatcacttccatATGCCGGGCAATCTGC

oLebron97_R	tgcaggatccgactcgagtgtcgacTCATGCGAACCAATCTCTCG
oLebron98_F	atgcggaggaatcacttccatATGTCGGATACCGAGAATCAGC
oLebron98_R	tgcaggatccgactcgagtgtcgacTCAATCATCAGCCGCCAAAATAGC
oLebron99_F	atgcggaggaatcacttccatATGATTGAGGTTTACAACTCGG
oLebron99_R	tgcaggatccgactcgagtgtcgacTCAGACGCACTCGGCG
oLebron100_F	atgcggaggaatcacttccatATGGAGTTCAAAGCTAATTGTTGC
oLebron100_R	tgcaggatccgactcgagtgtcgacTCAGCCCCTCTTGTCCTG
oLebron101_F	atgcggaggaatcacttccatATGGAGTTCGTGATGGAGTGC
oLebron101_R	tgcaggatccgactcgagtgtcgacTCACAGGACCATTGCAAAGAC
oLebron104_F	atgcggaggaatcacttccatATGACTGATGTTGATCCGATCTGG
oLebron104_R	tgcaggatccgactcgagtgtcgacTCAACTCTCATCAACCTCAGG
oLebron110_F	atgcggaggaatcacttccatATGTCTGAGGTTAAGAGCCTTTACG
oLebron110_R	tgcaggatccgactcgagtgtcgacTCACCCCGCCCCATTAG
oLebron111_F	atgcggaggaatcacttccatATGACGTTGTATCACCGGACG
oLebron111_R	tgcaggatccgactcgagtgtcgacTCACTTACGCTTCTTACGCGG
oLebron113_F	atgcggaggaatcacttccatATGCCCGCTTCAACGG
oLebron113_R	tgcaggatccgactcgagtgtcgacTCAGTCCGGTTCGATTCC
oLebron115_F	atgcggaggaatcacttccatATGACCTGTCAAATCCCGTGC
oLebron115_R	tgcaggatccgactcgagtgtcgacTCAGCAGTGCCCGCGTG
oLebron116_F	atgcggaggaatcacttccatATGGACCGGGTTGTGGTC
oLebron116_R	tgcaggatccgactcgagtgtcgacTCACTTGAGTGTGACGGTGG
oLebron117_F	atgcggaggaatcacttccatATGTACGGTAGAGGCATGAGC
oLebron117_R	tgcaggatccgactcgagtgtcgacTCAAGCGTCCCTGATGAGC
oLebron118_F	atgcggaggaatcacttccatATGCAGAGCGACAAATGCG
oLebron118_R	tgcaggatccgactcgagtgtcgacTCAACCCTGGTTTTTGCG
oLebron119_F	atgcggaggaatcacttccatATGGACTGCCTAGAAGGTCC
oLebron119_R	tgcaggatccgactcgagtgtcgacTCAGTCGTCCTCGTTCC
oLebron120_F	atgcggaggaatcacttccatATGCGCAAATTCATCAGCG
oLebron120_R	tgcaggatccgactcgagtgtcgacTCAGTTGGTGAGGCCG
oLebron121_F	atgcggaggaatcacttccatATGGACATGAGCAACGAAACG
oLebron121_R	tgcaggatccgactcgagtgtcgacTCAGCCCCCCATCTGC
oLebron122_F	atgcggaggaatcacttccatATGGTTTCTGACCCGACG
oLebron122_R	tgcaggatccgactcgagtgtcgacTCAAATCCGATCTTCAATGGAG
oLebron123_F	atgcggaggaatcacttccatATGCTGCCAAACATCCCAG
oLebron123_R	tgcaggatccgactcgagtgtcgacTCAGAAACCATGCTCGGAGAG
oLebron124_F	atgcggaggaatcacttccatATGGCCGCGTTCGCAAAG
oLebron124_R	tgcaggatccgactcgagtgtcgacTCAGCTGACGGCTTCTGC
oLebron125_F	atgcggaggaatcacttccatATGAAGCGCACACCGAAATG
oLebron125_R	tgcaggatccgactcgagtgtcgacTCATGCTGCTTCTCCTTCTGC

oLebron126_F	atgcggaggaatcacttccatATGGGCCGAGGTCGCG
oLebron126_R	tgcaggatccgactcgagtgtcgacTCACTTCTTAGCCCGGTC
oLebron127_F	atgcggaggaatcacttccatATGCGCGTAGGCACAC
oLebron127_R	tgcaggatccgactcgagtgtcgacTCACAGCAACCCAAGCTCG
oLebron128_F	atgcggaggaatcacttccatATGTCCGACATCCAGGC
oLebron128_R	tgcaggatccgactcgagtgtcgacTCAGGCCACCTTGCGC
oLebron129_F	atgcggaggaatcacttccatATGGTGAGAACAAAGATATACCCCG
oLebron129_R	tgcaggatccgactcgagtgtcgacTCAAGCGCAGGCCCCG
oLebron130_F	atgcggaggaatcacttccatATGAGCACCGCTATCCGC
oLebron130_R	tgcaggatccgactcgagtgtcgacTCAGACGGTTTCCAAAACG
oLebron131_F	atgcggaggaatcacttccatATGAGCACCACCCACACC
oLebron131_R	tgcaggatccgactcgagtgtcgacTCAGCGTGCGTACTGC
oLebron132_F	atgcggaggaatcacttccatATGGGTGTGTGTTTGCTGG
oLebron132_R	tgcaggatccgactcgagtgtcgacTCAAGCCAGTGCGCCTAAAG
pExTra_seqF	GTACCCGTGTGTACGACCAGC
pExTra_uniR	CCCTTCGAGACCATAGATCTGTTCC
oLebron4i_F	ggctggtactgaggattcg
oLebron5i_F	ggcgaggtcgagtatctgg
oLebron16ia_F	aagaggctatcgctgagg
oLebron16ib_F	acaatgacggtcgtcttg
oLebron16ic_F	agctccgttcggagattgatg
oLebron16id_R	ccggcattcaccgcatcc
oLebron16ie_R	gtgaccagcaatcgactgg
oLebron18i_F	ggattggcagtgtttcg
oLebron20ia_F	ttggtgtcggggccgaacg
oLebron20ib_F	agtcggcgatcacgttggc
